# Lineage-Specific Expansion of IFIT Gene Family: An Insight into Coevolution with IFN Gene Family

**DOI:** 10.1371/journal.pone.0066859

**Published:** 2013-06-20

**Authors:** Ying Liu, Yi-Bing Zhang, Ting-Kai Liu, Jian-Fang Gui

**Affiliations:** State Key Laboratory of Freshwater Ecology and Biotechnology, Institute of Hydrobiology, Chinese Academy of Sciences, Wuhan, China; University of Lausanne, Switzerland

## Abstract

In mammals, IFIT (Interferon [IFN]-induced proteins with Tetratricopeptide Repeat [TPR] motifs) family genes are involved in many cellular and viral processes, which are tightly related to mammalian IFN response. However, little is known about non-mammalian IFIT genes. In the present study, IFIT genes are identified in the genome databases from the jawed vertebrates including the cartilaginous elephant shark but not from non-vertebrates such as lancelet, sea squirt and acorn worm, suggesting that IFIT gene family originates from a vertebrate ancestor about 450 million years ago. IFIT family genes show conserved gene structure and gene arrangements. Phylogenetic analyses reveal that this gene family has expanded through lineage-specific and species-specific gene duplication. Interestingly, IFN gene family seem to share a common ancestor and a similar evolutionary mechanism; the function link of IFIT genes to IFN response is present early since the origin of both gene families, as evidenced by the finding that zebrafish IFIT genes are upregulated by fish IFNs, poly(I:C) and two transcription factors IRF3/IRF7, likely via the IFN-stimulated response elements (ISRE) within the promoters of vertebrate IFIT family genes. These coevolution features creates functional association of both family genes to fulfill a common biological process, which is likely selected by viral infection during evolution of vertebrates. Our results are helpful for understanding of evolution of vertebrate IFN system.

## Introduction

The innate immune response to viral infection is largely dependent on host production of interferons (IFNs) that are a family of cytokines with antiviral, antiproliferative and immunomodulatory properties [1], [2]. The multiple effects of IFNs are mediated through induction of a large array of IFN-stimulated genes (ISGs), which are regulated predominantly by Jak-Stat (Janus kinase-signal transducer and activator of transcription) signaling pathway [2]. IFN-activated signaling enables formation of a transcription factor complex ISGF3 (IFN-stimulated gene factor 3), which subsequently translocates to nucleus and directly binds to the IFN-stimulated response element (ISRE) in the promoters of ISGs triggering gene transcription [2]. Consistently, some ISGs, such as PKR [3], [4], PKZ [3], [4] and IFI56 [5], , have shown abilities to affect viral replication, transcription and cell growth.

IFI56 (IFN-induced protein 56 kDa, also called ISG56) is the first ISG to be discovered and cloned [10], due to its dramatically up-regulated transcriptional and translational level in IFN-treated cells relative to no or very weak expression in normal cells [11], [12]. Subsequent studies reveal that IFI56 belongs to a well-conserved gene family, named IFIT (IFN-induced proteins with Tetratricopeptide Repeat (TPR) motifs) family [13]. In addition to IFN treatment, other IFN stimuli including virus infection, double-stranded RNA and lipopolysaccharicdes also strongly induce the expression of these family genes, implying pivotal roles under diverse cellular stresses [9], [13], [14], [15], [16], [17], [18]. A structural hallmark of IFIT proteins is that they all contain multiple TPR motifs dispersed throughout the whole sequences [1], [13], [19]. The TPR motif is a degenerate thirty-four amino acid residue repeat unit involved in protein-protein interaction and assembly of large protein complexes [1]. The possession of multiple TPR domains is believed to endow IFIT proteins on a multitude of effects on cellular and viral functions, including regulation of transcription, translation, antiproliferative effects and also negative regulation of host inflammatory and antiviral response [20], [21]. Recently IFIT family members have been proven for selectively restricting virus replication by recognition of viral mRNA lacking 2′-O methylation or with a 5′-triphosphate RNA, likely through its TPR motifs [8], [22].

Genomic structure analyses of mammalian IFIT family genes reveal a common evolutionary origin possibly by gene duplication. The first evidence is that four human IFIT members, including IFI56/IFIT1, IFI54/IFIT2, IFI60/IFIT3 and IFI58/IFIT5, are tandem located in a locus on chromosome 10 and consistently, three mouse homologues of IFIT members are also clustered together although loss of IFI58 homologue [20]. The second is that all mammalian IFIT proteins are classified into four subgroups with a clear orthologous relationship [1], [13], [19]. Finally, most of these mammalian genes show a similar exon/intron organization with two exons separated by an intron of few kilobases in length, the first one encoding only the ATG start codon and two nucleotides of the second codon plus 5′ UTR, and the second encoding the rest of the mRNA [23], [24]. In addition, these genes possess a similar promoter structure characterized by two or three ISRE motifs normally located within 200 bp upstream of the TATA box [16], [23], [24], [25]; the ISRE motif is responsible for IFIT gene transcription by binding to the transcriptional complex ISGF3 in an IFN-dependent manner or to transcription factors such as IRF3/IRF7 in an IFN-independent manner [16], [18], [25], [26].

Fish IFIT-like genes have recently been identified from crucian carp (*Carassius auratus*), showing a low level of identity and similarity to their mammalian homologues [19]. However, the evolutionary origin and biological significance of IFIT family remains elusive. Fish appear to possess a complete IFN system [27], evidenced by the facts that fish genomes contain multiple IFN genes in a tandem arrangement [28], [29], and that these IFN genes exerts antiviral effects by different receptors [30], but by a similar Jak-Stat pathway using the same transcription factors [31], [32], [33], [34]. Like mammalian homologues, crucian carp IFIT-like genes possess similar expression patterns, with a silent expression in mock-induced fish cell lines but significant upregulation by either viral infection or crucian carp IFN treatment [19], [31], [34], [35].Considering the fact that the action of IFNs relies on the IFN-inducible proteins, it is worthwhile to explore the genomic structure of IFIT family genes and their expression link to IFNs.

In the present study we reported large-scale search for IFIT genes from vertebrate and invertebrate genome databases. Comprehensive analyses of genomic structure and phylogenetic trees indicated that the evolutionary origin of IFIT gene family is temporally associated with the appearance of jawed animals and that the family members are involved in an active expansion by lineage-specific duplication. Similar to human and mouse IFIT members [16], [23], [24], [25], the ISRE motif is also present in the promoters of non-mammalian vertebrate IFIT genes, which are ascribed to IFN induction of zebrafish IFIT genes. Interestingly, vertebrate IFN family genes seem to show a common evolutionary origin and a similar expansion fashion. These results together suggested that the features of IFIT gene family linking to IFN response are conserved from fish to mammals.

## Results

### Predication and Annotation of IFIT Family Genes

**Figure 1:**
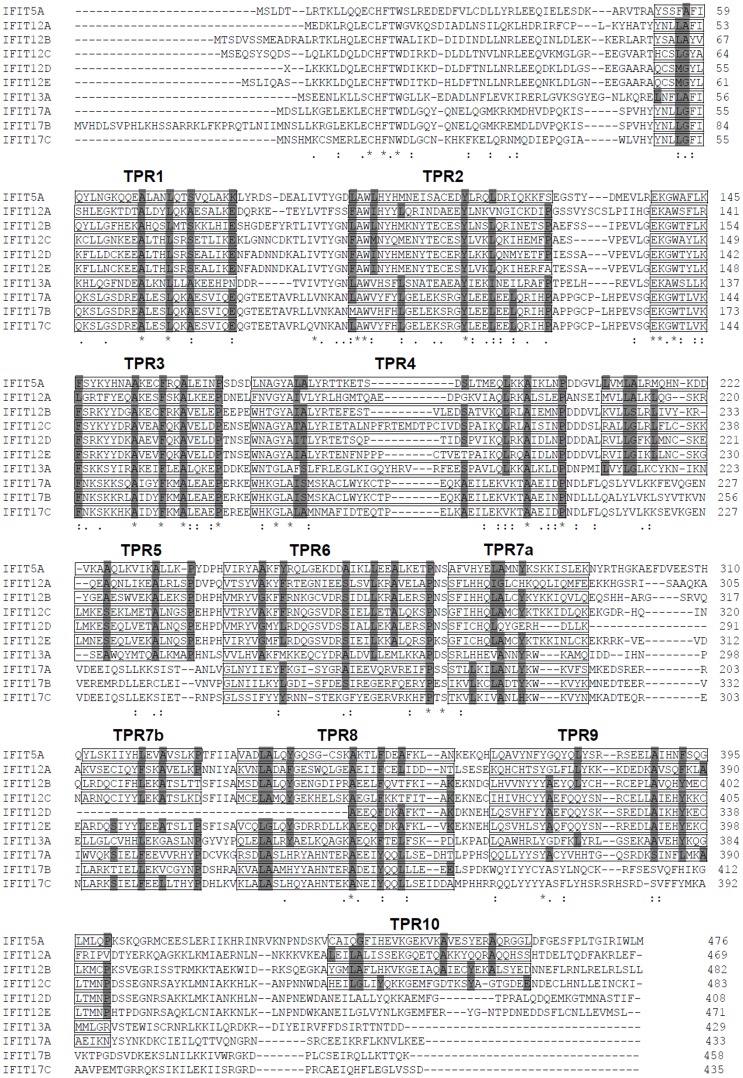
Sequence alignments of zebrafish IFIT proteins with Clustal W (1.8) program. Zebrafish IFIT family genes were identified by BLAST search of zebrafish genome. The putative TPR motifs were indicated by boxes, according to the rule that each motif should contain more than 4 conserved residues of the 8 key sites within the consensus sequences of TPR motifs summarized by NCBI. Identical (*) and similar (. and :) residues are indicated.

Four human (*H. sapiens*) IFIT genes and three mouse (*Mus musculus*) IFIT genes have been well-characterized [20]; therefore, extensive BLAST analyses based on sequence homology with these IFIT proteins were used to search IFIT genes from the genome databases of vertebrates including ten mammalian species, two avian species, one reptilian species, one amphibian species and six fish species (Table 1). Considering a low level of sequence identity among distant species, two fish IFIT proteins identified previously, crucian carp IFI56 (IFIT1) and IFI58 (IFIT5) [19], were also included as query sequences. 42 mammalian genes and 10 zebrafish (*D. rerio*) genes, which have been annotated in genome databases, were easily identified as IFIT genes (Table 1). Subsequently, these 52 annotated genes were further used to do BLAST search followed by GENSCAN. Additional 53 IFIT-like genes and 2 pseduogenes homologous to human IFIT1P from chimpanzee (*P. troglodytes*) and rhesus monkey (*M. mulatta*) were identified, 49 of which were identified by GENSCAN (Table S1). All vertebrate species studied contain at least one (Table 1). These putative proteins share at least 18% (three-spinned stickleback IFIT-F) up to 94.7% (chimpanzee IFIT1) identities to human IFIT1 protein (Table S2), span 160–773 amino acids in length, and harbor at least two up to ten predicated TPR motifs throughout the whole amino acid sequences (Table S1). For example, ten zebrafish homologous genes encode proteins of 429 to 773 amino acids in length and with 7 to 10 potential TPR motifs (Figure 1).

**Table 1:** Statistics for IFIT homologous gene search from databases.

Classification	Species	Gene number and location	Methods
*Echinoderms	Sea urchins *S. purpuratus*	No identifiable genes	GENSCAN
*Hemichordates	acorn worms *S. kowalevskii*	3 candidate genes; 1(scaffold49422), 1(scaffold43933), 1(scaffold3509)	GENSCAN
*Cephalochordates	Amphioxi *B. floridae*	3 candidate genes; 1(scaffold459), 1(scaffold1321), 1(scaffold159)	GENSCAN
*Urochordates	sea squirts *C. intestinalis*	No identifiable genes	
*Cyclostomes	Hagfish *E. burgeri*	1; EST(BJ653783.1)	
	Lampreys *P. marinus*	3; ESTs (FD701002.1, CO543267.1, CO548223.1 )	
Cartilaginous fish	elephant shark *C. milii*	8 (unknown)	GENSCAN
Bony fish	Zebrafish *D. rerio*	10; 1(Ch5), 5(Ch12),1(Ch13), 3(Ch17)	Annotated
	medaka *O. latipes*	10; 6(Ch4), 4(unknown)	GENSCAN
	torafugu *T. rubripes*	1; 1(Scaffold 622)	GENSCAN
	Three-spinned stickleback *G. aculeatus*	10; 9(groupVIII), 1(unknown)	GENSCAN
	Tetraodon *T. nigroviridis*	2; 2(unknown)	GENSCAN
Amphibians	Western clawed frog *X. tropicalis*	11; 8(GL172907.1), 2(GL181739.1), 1(GL180711.1)	GENSCAN
Reptiles	Anole Lizard *A. carolinensis*	2; 2(Unknown)	GENSCAN
Birds	chickens *G. gallus*	1; 1(Ch6)	Annotated
	Zebra Finch *T. guttata*	2; 1(Ch6), 1(LEG22)	Annotated&GENSCAN
Mammals	Human *H. sapiens*	6; 5(Ch10), 1(pseudogene,Ch13)	Annotated
	Chimpanzee *P. troglodytes*	6; 5(Ch10), 1(pseudogene,Ch13)	Annotated&GENSCAN
	Rhesus monkey *M. mulatta*	5; 4(Ch9), 1(pseudogene,Ch17)	Annotated&GENSCAN
	Mouse *Mus musculus*	4; 4(Ch19)	Annotated
	Rat *Rattus norvegicus*	4; 4(Ch1)	Annotated
	Cattle *Bos Taurus*	4; 4(Ch26)	Annotated
	Horse *Equus caballus*	3; 3(Ch1)	Annotated
	Pig *Sus scrofa*	4; 4(Ch14)	Annotated
	Dog *C. familiaris*	5; 4(Ch4), 1(Ch28)	Annotated
	Platypus *O. anatinus*	3; 3(Unknown)	Annotated&GENSCAN

* The genes identified in these species exhibit the closest BLAST hit with E value <10^−6^, but are not real IFIT family genes by phylogenetic tree analysis.

**Figure 2:**
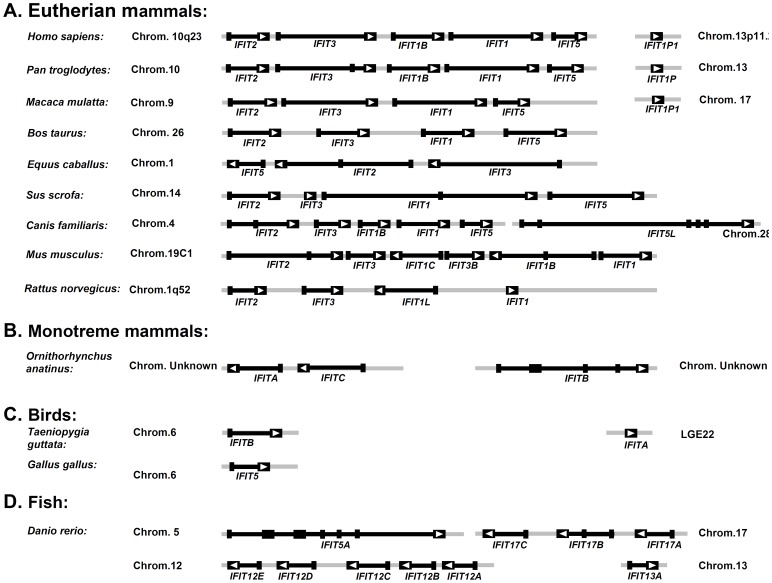
Gene organization of IFIT loci from mammals, birds and zebrafish. The exon/intron structures of eutherian mammals (A), monotreme platypus (B), birds (C) and zebrafish (D) were compared. Chimpanzee and rhesus monkey IFIT1P genes that are located away from the main IFIT loci, and Zebra Finch IFITA residing in other scaffold, were identified by GENSCAN. The exons are indicated by black boxes, and the introns by grey lines. The orientation of the open reading frames is indicated by arrowheads.

All mammalian species available contain four members of IFIT family, including IFIT1, IFIT2, IFIT3, and IFIT5, with the exception of both rat (*R. norvegicus*) and mouse lacking IFIT5, and horse (*E. caballus*) lacking IFIT1 (Figure 2A). In addition, several IFIT-like genes, obviously generated by duplication of IFIT1 or IFIT3, have been identified in human, chimpanzee (*P. troglodytes*), dog (*C. familiaris*), mouse and rat. These genes are tandem located in a common chromosome except for the pseudogenes IFIT1Ps of three primate species and dog IFIT5L, which reside in separate chromosomes (Figure 2A). Three IFIT genes have also been identified in platypus (*O. anatinus*), a monotreme mammalian species exhibiting a fascinating combination of reptilian and mammalian characters [36] (Figure 2B).

In contrast to a relatively constant number of IFIT family genes in mammals, there are varied gene copies in birds, reptiles, amphibians, and fish (Table 1). For example, two IFIT genes were identified in Zebra Finch (*T. guttata*) but only one in chicken (*G. gallus*) (Figure 2C); a total of 11 homologues were found in western clawed frog (*X. tropicalis*). Fish genomes also contain varied IFIT-like genes, with one in torafugu (*T. rubripes*) but ten in zebrafish, three-spinned stickleback (*G. aculeatus*) and medaka (*O. latipes*) (Table 1). Since zebrafish genome assembly is relatively complete compared to the other fish, zebrafish IFIT gene family is further analyzed. Ten zebrafish genes are distributed in four chromosomes, with five in chromosome 12 (IFIT12A, IFIT12B, IFIT12C, IFIT12D and IFIT12E), three in chromosome 17 (IFIT17A, FIT17B and IFIT17C), one in chromosome 5 and one in chromosome 13 (IFIT5A and IFIT13A) (Figure 2D). Elephant shark (*C. milii*), a cartilaginous fish that is the phylogenetically oldest species of living jawed vertebrates and is not believed to experience the whole-genome duplication event [37], also contains eight homologous genes (Table 1).

Finally, extensive BLAST search of invertebrate genome databases hit many TPR-containing proteins, and three in acorn worm (*S. kowalevskii*) and Cephalochordata species lancelet (*B. floridae*) were selected since they showed the closest BLAST hit with E-value<10^−6^. According to the same criteria, three genes are identified in sea lamprey (*P. marinus*) and one in hagfish (*E. burger*) by BLAST search of EST and protein databases (Table 1). However, these TPR-containing proteins show no significant identity to the known IFIT proteins (Table S2).

### Conserved Genomic Organization of IFIT Family Genes

The exon/intron organization of IFIT genes in ten mammalian species, two avian species and zebrafish (up to 60 genes) were further analyzed. Due to lack of mRNA or EST information, the genomic structures of IFIT genes in green anole, western clawed frog and the remaining five fish species were unavailable. As shown in Figure 2, 46 out of 60 genes share a similar genomic structure with two exons that are separated by an intron, 7 genes with three exons (horse IFIT2, pig IFIT1, dog IFIT2, mouse IFIT2 and IFIT1B, zebrafish IFIT17B), and 3 genes with more than 5 exons (dog IFIT5L, platypus IFITB and zebrafish IFIT5A). Both pig IFIT3 and Zebra Finch IFITA are encoded by only one exon, similar to pseudogene IFIT1P in the three primate species; however, both genes are indeed functional genes, as supported by EST or mRNA information (Table S1).

The gene arrangement of IFIT loci was next analyzed. IFIT gene cluster in most mammalian lineages is composed of four members, if existed, in the same order of IFIT2-IFIT3-IFIT1-IFIT5 and with the same transcriptional orientation (Figure 2A). An exception is horse genome that appears to lack IFIT1, and displays a different gene arrangement with IFIT3, IFIT2 and IFIT5 orderly. Compared to rhesus monkey (*M. mulatta*), a gene duplication event occurred within IFIT loci of human and chimpanzee, which gave a birth of IFIT1B paralogous to IFIT1 genes. The same is true for IFIT loci of dog, mouse and rat (Figure 2A). Intriguingly, compared to rat, an additional gene duplication event occurred in mouse IFIT locus, which resulted in generation of a novel IFIT3B-IFIT1B segment with reference to the IFIT3-IFIT1C-containing fragment. It is not sure whether the IFIT genes from platypus and Zebra Finch reside on one chromosome due to incomplete genome assembly (Figure 2B and 2C). In zebrafish, ten IFIT genes reside in four different chromosomes; the genes in the same cluster bear the same transcriptional orientation, as seen in mammals (Figure 2D).

**Figure 3:**
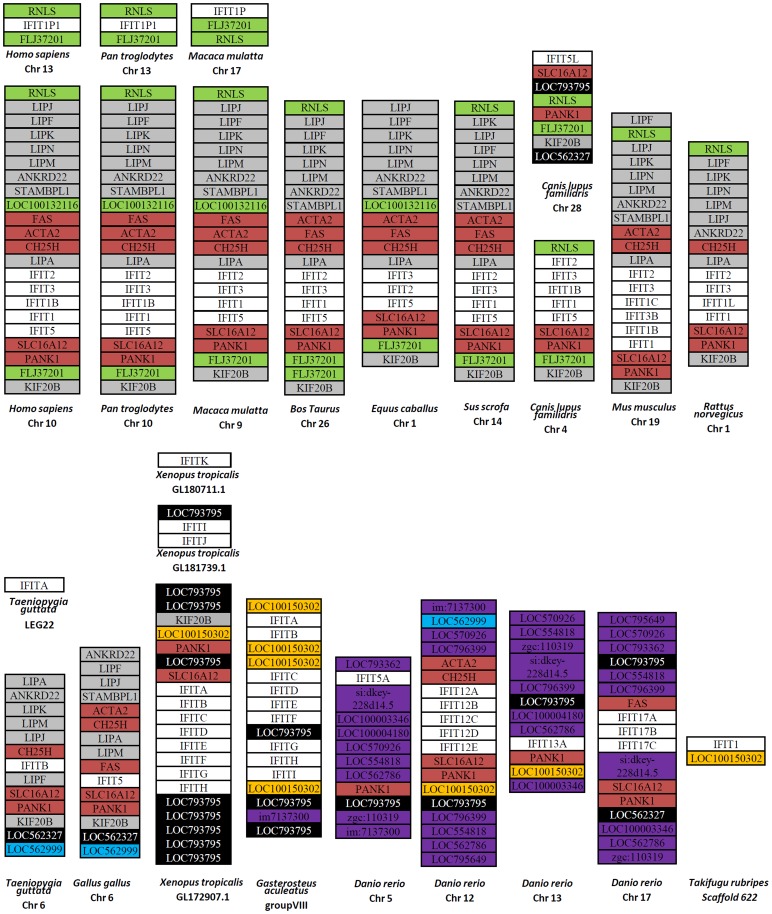
Comparative analyses of gene synteny of vertebrate IFIT loci. Approximately 10-million-bp DNA sequences flanking IFIT loci of the indicated species were analyzed for gene colinearity. For dog Chromosome 4, Zebra Finch LEG22, western clawed frog GL180711.1, GL181739.1, and torafugu Scaffold 622, only 1170 kb, 195 kb, 25 kb, 12 kb and 7 kb were analyzed due to the incomplete genome information. The surrounding genes, if needed, were predicated by GENSCAN and compared by local blast software. Only the conserved flanking genes were shown. The red surrounding genes are conserved in all vertebrate lineages. The black is seen in birds, amphibians, fish and dog. The grey is conserved in mammals, birds and amphibians. The blue is in birds and fish, the yellow in fish and amphibians, the purple exclusively in fish lineages, and the green only in mammalian lineages.

Finally, approximate 10 million base pairs of genome sequences flanking IFIT locus were analyzed to investigate whether the surrounding genes are evolutionarily conserved. As shown in Figure 3, mammalian lineages show a good gene synteny, with the conserved gene arrangement of more than 10 genes upstream and at least 3 genes downstream of IFIT locus. Most of the surrounding genes also appear in two birds (Zebra Finch and chicken), and some of them in amphibians (western clawed frog), although in a slightly distinct gene arrangement (indicated by grey in Figure 3). In addition, all the genes surrounding bird IFIT loci can be found either in mammals or in amphibians and fish. Five surrounding genes are common in all vertebrate lineages available. They are FAS, ACTA2, CH25H, SLC16A12 and PANK1 (indicated by red). These genes are not seen in the flanking regions of both singleton genes western clawed frog IFITK and Zebra Finch IFITA, but two of them, SLC16A12 and PANK1, appear in the flanking region of dog IFIT5L. These results indicated that the gene synteny of IFIT loci is well conserved from fish to mammals.

Compared to mammals, fish exhibit less conservation of the surrounding genes, with diverse gene orders in different species and even in different chromosomes of the same species, which is possibly ascribed to a rapider evolution during radiation of teleost species than is seen in mammalian lineages [38]. For example, four common surrounding genes described above are found in the flanking region of IFIT locus in zebrafish chromosome 12 and three in chromosome17, but only one in chromosome 13 and chromosome 5, respectively (indicated by red). Most surrounding genes are found exclusively in fish lineages (indicated by purple). Notably, three-spinned stickleback has a discontinuous IFIT locus separated by genes LOC10015032 and LOC793795, both of which appear in frog and the latter of which also in birds, dog and the other fish species (Figure 3).

### Lineage-specific Expansion of IFIT Gene Family and IFN Gene Family

**Figure 4:**
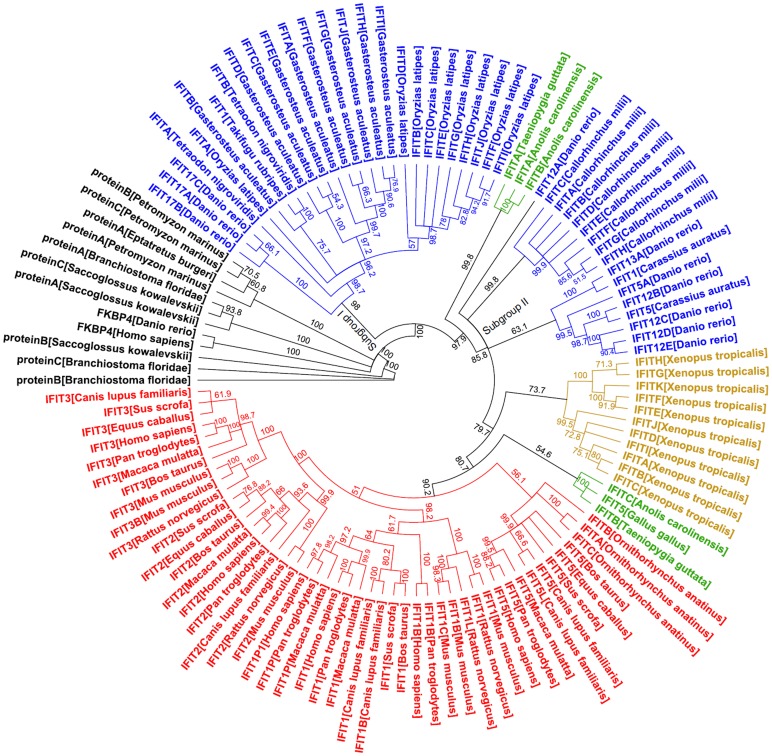
Phylogenetic analyses of IFIT family proteins identified in this study. A neighbor-joining tree was constructed based on analysis of 107 IFIT family protein sequences using Geneious Pro 5.4.6 program, with bootstrap values for 1000 replicates. Human FKBP4 and zebrafish FKBP4 as well as the TPR-containing proteins identified from acorn worm, lancelet, hagfish and sea lamprey were included, indicating that these proteins are not real IFIT homologues (indicated by black). There are long C-terminus of zebrafish IFIT5A (from position 509 to 773 amino acid) and N-terminus of dog IFIT5L (from 1 to 245 amino acid), which were not conserved and then were deleted to exclude disturbance for multiple alignments. The blue represented IFIT genes from fishes, the green from birds and reptiles, the yellow from amphibians, and the red from mammals.

Phylogenetic trees were constructed to investigate the evolutionary history of IFIT gene family. Human and zebrafish peptidyl-prolyl cis-trans isomerase FKBP4 are included as outgroup since they are also TPR-containing proteins. A neighbor joining phylogenetic tree showed extensive expansion of IFIT gene family by lineage-specific and species-specific duplication (Figure 4). A similar result was obtained by maximum likelihood method (Figure S1). Consistent with sequence identity analyses (Table S2), TPR-containing proteins from hagfish, sea lamprey, lancelet and acorn worm are grouped well with two FKBP4 proteins, which are obviously distant from the branch composed of vertebrate IFIT proteins (Figure 4). Thus, these non-jawed vertebrate proteins with TPR motifs are not IFIT counterparts.

As shown in Figure 4, all IFIT proteins are classified into four independent groups: mammals, birds together with reptiles, amphibians, and fish. In mammalian lineages, IFIT proteins form 4 subgroups: IFIT1s, IFIT2s, IFIT3s and IFIT5s. Obviously, those IFIT-like proteins generated by segmental duplication are paralogous to their counterparts, such as IFIT1B/C to IFIT1, IFIT3B/C to IFIT3, and IFIT5L to IFIT5. Particularly, platypus ITIFA and ITIFC are paralogous to each other, and platypus ITIFB is as closely related to the mammalian IFIT5 subgroup as it is to the platypus ITIFA and ITIFC genes. In fish, two subgroups are formed. Subgroup I comprises proteins from the more advanced fish species such as two pufferfishes, three-spinned stickleback and medaka, and subgroup II from the more primitive fish species including elephant fish and crucian carp. Interestingly, three zebrafish proteins on chromosome 17 (IFIT17A/B/C) are clustered with subgroup I, and the remaining seven zebrafish proteins clustered with subgroup II, indicating a possibility of loss of subgroup II in the more advanced fish species. Some fish members show a one-to-one orthologous relationship, such as between zebrafish IFIT13A and crucian carp IFIT1, zebrafish IFIT12B and crucian carp IFIT5, and among spotted green pufferfish IFITB, torafugu IFIT1, and three-spinned stickleback IFITB. Remarkably, the proteins from reptile and birds are clustered together, thus classified into two subgroups: one related to fish group, and the other to mammalian group. These results suggest that lineage-specific and species-specific duplication have contributed to extensive expansion of vertebrate IFIT family.

IFN-activated expression of mammalian IFIT proteins arouses our interest to investigate the origin of IFN gene family. Consistent with a previous result [39], IFN homologues were not found in lancelet genome. BLAST search of EST and protein databases did not find any sequences homologous to IFNs in sea lamprey and hagfish. However, we identified three IFN homologous genes from elephant shark genome by GENSCAN. Consistent with previously phylogenetic tree analyses [29], [40], [41], IFN genes are grouped together based on different lineages (Figure S2). These results indicated that vertebrate IFN gene family, similar to IFIT gene family, has expanded through lineage-specific gene duplication.

### IFN Induction of Fish IFIT Family Genes

**Figure 5:**
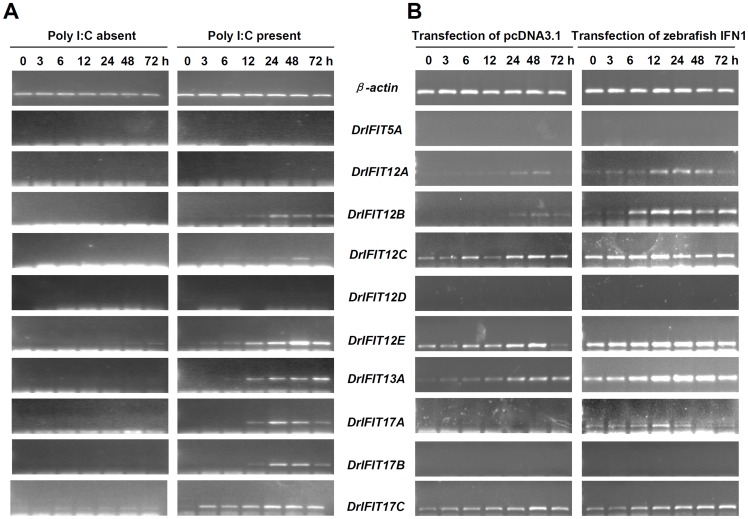
RT-PCR detection of zebrafish IFIT genes in response to poly(I:C) and IFN. ZFL cells seeded in 6-well plates were transfected with poly(I:C) (4 µg/well, final concentration 4 µg/mL) or with PBS as control (A), and ZF4 cells seeded in 6-well plates were transfected with zebrafish IFN1 construct (4 µg/well, final concentration 4 µg/mL) or with pcDNA3.1 as control (B). At the indicated time points, the treated cells were extracted for detection of IFIT mRNAs.

The results above suggested that vertebrate IFIT genes and IFN genes originated from an ancestor of fish and mammals; therefore, whether fish IFIT gene expression was induced by IFN was further concerned. In initial assays, RT-PCR was used to detect IFIT gene transcripts in cultured zebrafish cells ZFL when transfected with poly(I:C), an effective IFN stimulus [31]. Compared to no or weak expression in mock-transfected cells, seven of ten zebrafish IFIT genes were significantly induced by poly(I:C); for the other three genes (IFIT5A, IFIT12A and IFIT12D), no expression was detected in either control or transfected cells (Figure 5A). In subsequent assays, another cultured zebrafish cells ZF4 were transfected with zebrafish IFN1 construct. It showed no transcripts detected for IFIT5A, IFIT12D and IFIT17B, but the other gene expression was significantly induced (Figure 5B). In addition, zebrafish IFIT genes displayed a relative higher level of basal expression in normal ZF4 cells than seen in ZFL cells, including IFIT12A, IFIT12B, IFIT12C, IFIT12E, IFIT13A and IFIT17C (Figure 5). These results together suggested that fish IFIT genes can be induced by IFN or IFN stimulus although cell type-specific induction was observed.

### Conserved Promoter Structure of IFIT Genes Contributing to IFN Induction

**Figure 6:**
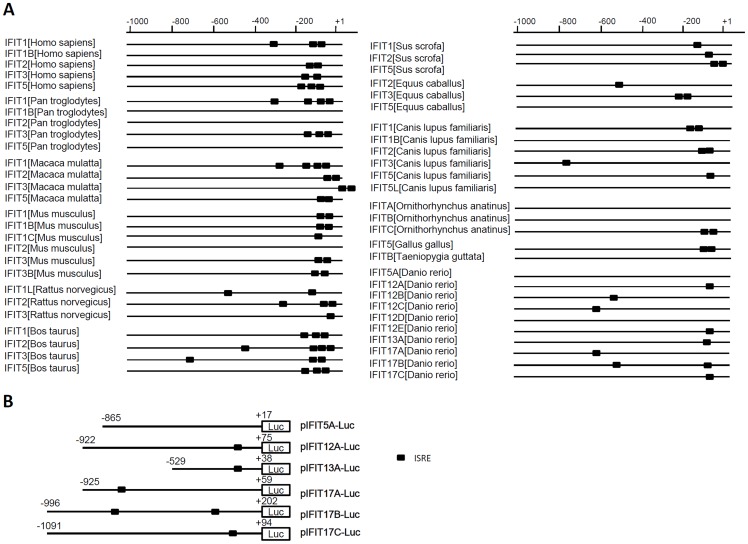
Comparative analyses of ISRE-containing promoters of IFIT family genes. (A) More than 1 kb of 5′ flanking sequences upstream of transcription start sites of IFIT genes from human, chimpanzee, rhesus monkey, mouse, rat, horse, pig, cattle, dog, chicken, Zebra Finch, and zebrafish were searched for ISRE motifs by Geneious and TFSEARCH combined with manual inspections. ISRE motifs are indicated by black rectangles and the numbers above show the sequence distances from the transcription start sites. (B) Schematic representations of six zebrafish IFIT gene promoter-driven luciferase constructs.

Human and mouse IFIT genes respond to IFN or IFN stimuli through the ISRE motifs present in their promoters [16], [18], [25]. We analyzed 5′ flanking region of IFIT genes that are available from genome databases. Generally, IFIT gene promoters from mammalian species possess two or more ISRE motifs (Figure S3), which are generally located within 200bp upstream of the transcriptional start site and in the same orientation, rarely in the distal region of promoters, such as in human IFIT1 gene (Figure 6A). However, no typical ISRE motif is found in the promoters of chimpanzee IFIT2, IFIT1B from human, chimpanzee and dog, IFIT5 from chimpanzee, horse and dog. In zebrafish, apart from IFIT5A and IFIT12D whose promoters did not contain typical ISRE motifs, the other 8 genes bear at least one within ∼800 bp upstream of the transcriptional start site (Figure 6A).

**Figure 7:**
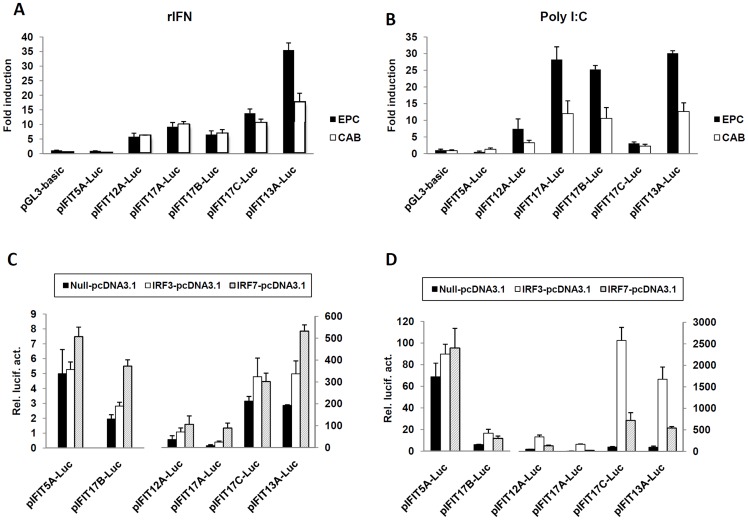
Activation of zebrafish IFIT promoters by recombinant IFN, Poly I:C and IRF3/IRF7. (A) EPC or CAB cells seeded in 24-well plates were cotransfected with 0.25 µg of zebrafish IFIT promoter-driven luciferase constructs and 0.025 µg of pRL-TK. 24 h post transfection, cells were treated by recombinant IFN (20 ng/ml) for another 24 h, and then harvested for detection of luciferase activity. The fold induction was normalized by the luciferase activity from control cells. (B) EPC or CAB cells seeded in 24-well plates were cotransfected as in (A). 24 h post transfection, cells were transfected again with 4 µg/ml poly(I:C) for another 24 h, then harvested for detection of luciferase activity. (C and D) EPC (C) and CAB cells (D) seeded in 24-well plates were cotransfected as in (A) with the indicated plasmids. 48 h post transfection, cells were harvested for detection of luciferase activity. Luciferase activities were presented as relative light units (RLU) normalized to the amount of Renilla luciferase activities.

To determine the role of ISRE motif in responsiveness of fish IFIT genes to IFN, 6 zebrafish IFIT gene promoters are cloned to make promoter-driven luciferase constructs (Figure 6B). We hypothesized that these zebrafish gene promoters except IFIT5A promoter might be activated by IFN, since all but IFIT5A promoter contain ISRE motifs. These promoter constructs were transfected into EPC cells followed by treatment with recombinant IFN. As expected, IFN treatment did not lead to an increase in zebrafish IFIT5A promoter-driven luciferase activity compared to the control cells treansfected with empty vector pGL3; however, significant luciferase activities were detected for the other five fish promoters by 3 to 30 folds (Figure 7A). When it came to poly(I:C) transfection, about 5∼31 fold luciferase activities were observed for these five promoters but no appreciable increase for IFIT5A promoter (Figure 7B). Transfection of CAB cells with the six constructs obtained similar results (Figure 7A and 7B). In addition, there were no appreciable differences in sensitiveness to IFN or poly(I:C) between distal ISRE-containing promoters (IFIT17A and IFIT17B) and proximate ISRE-containing promoters (IFIT12A, IFIT13A and IFIT17C). These results suggested that ISRE motifs contribute to the induction of fish IFIT genes by IFN or poly(I:C).

In mammals, IRF3 directly induced the expression of human IFI56 through binding to ISRE motifs without the need of IFNs [26]. Thus, the activation of zebrafish IFIT gene promoters by IRF3 or IRF7 was investigated. As shown in Figure 7C, overexpression of zebrafish IRF3 or IRF7 in EPC cells resulted in significant activation of the promoters of IFIT12A, IFIT13A, IFIT17A and IFIT17C, and IRF3 showed a higher ability to activate fish promoters than IRF7. When CAB cells were transfected, a similar result was seen, but with a higher inducibility of IRF7 than IRF3 (Figure 7D). However, under the same conditions, IFIT5A promoter and IFIT17B promoter were not activated by IRF3 or IRF7 (Figure 7C and 7D).

## Discussion

The structure hallmark of IFIT proteins are multiple TPR domains that are dispersed in the entire protein sequences [13]. Besides IFIT proteins, there are other TPR-containing proteins ubiquitously distributed in all kinds of eukaryotic organisms. Using vertebrate IFIT proteins as query sequences, BLAST search would find many TPR-containing proteins in invertebrate genome databases. In the present study, some TPR-containing proteins from invertebrates including acorn worm and lancelet, jawless vertebrates including hagfish and lamprey exhibit the closest BLAST hit with E value<10^−6^(Table 1), but do not belong to IFIT family by phylogenetic analyses (Figure 4). This is likely due to that TPR is a structural motif composed of a degenerate 34 amino acid unit [1], [13]. In contrast, IFIT homologous genes exist in all jawed animal including elephant shark, the phylogenetically oldest species of living jawed vertebrates [37]. Thus, the evolutionary origin of IFIT gene family is temporally associated with the appearance of jawed animals, possibly since divergence of jawless vertebrates and jawed vertebrates.

The data in the present study reveal extensive expansion of vertebrate IFIT genes by lineage-specific duplication, as evidenced by formation of different clades based on different vertebrate lineages and by conserved gene colinearity of IFIT loci (Figure 3 and Figure 4). In addition, species-specific duplication is also seen. For example, different fish species have varied number of IFIT members (Figure 3), most of which seemed to be generated after radiation of teleosts (Figure 4). Zebra Finch IFITA gene is located away from IFIT locus, without introns and the conserved neighboring genes (Figure 2 and Figure 3), possibly generated by a reverse transcriptase-generated copying mechanism (retrotransposition). However, dog IFIT5L arose likely through chromosome segmental duplication followed by interchromosome rearrangement, since it is paralogous to dog IFIT5 and displays good gene synteny with IFIT locus (Figure 3).

The species-specific duplication reveals an active creation of IFIT genes, particularly in fish lineages. There are varied IFIT gene copies located in different zebrafish chromosomes, and even in the same chromosome, the IFIT locus of three-spinned stickleback is interrupted by flanking genes (Figure 3). This is likely due to rapider gene duplication events and a higher rate of chromosomal rearrangements during evolution of teleost lineages than the other vertebrates [38]. More recent gene duplication events were also found in mammalian lineages. For example, pseudogene IFITP1 is found exclusively in the three primates, indicating that a gene duplication event took place about 75 Myr ago since divergence of primates and rodents [42]. Existence of IFIT1B in both human and chimpanzee but not in rhesus monkey might be due to a gene duplication event after divergence from rhesus monkey but before split of human and chimpanzee. Similarly, a recent chromosome segmental duplication led to appearance of IFIT3B and IFIT1C genes in mouse genome after divergence of mouse and rat about 12–24 Myr ago. In addition, the lack of IFIT1 in horse and IFIT5 in rodents might imply occurrence of gene loss events during evolution.

The phylogenetic analyses provide valuable clues to explain the evolutionary history of vertebrate IFIT gene family. Compared to elephant shark, additional whole genome duplication (WGD) occurred around 450 Myr ago [38], which might lead to IFIT gene duplication in teleost ancestor. Consistently, elephant shark contains only one subgroup of IFIT members (subgroup II). It is likely that the more primitive fishes including zebrafish should have two (subgroup I and subgroup II); however, the more advanced fishes, such as medaka, three-spinned stickleback and two pufferfishes, have retained only subgroup I of IFIT members, likely due to loss of subgroup II when they diverged later from the more primitive fishes. Similarly, the amphibian ancestor acquired an ancestral gene of IFIT subgroup II since divergence from the tetrapods about 360 Myr ago, which subsequently underwent lineage-specific expansion leading to a subfamily of amphibian genes. It is believed that the extant mammals, birds and reptiles possess a common amniotes ancestor, which split into sauropsids (leading to birds and reptiles) and synapsids (leading to mammal-like reptiles) around 315 Myr ago [36]. Monotremes (platypus) diverged from the therian mammal lineage about 166 Myr ago [43], and reptile (green annole) from bird about 280 Myr [44]. Thus, we address a hypothesis for the evolution of IFIT gene family. About 315 Myr ago, the ancestral gene related to fish group was inherited and subsequently duplicated leading to a new ancestral gene in an amniotes ancestor. Both types of ancestral genes were inherited to the diapsids ancestor about 280 Myr ago. The subsequent lineage-specific gene duplication resulted in existence of two subgroups of IFIT members in the extant birds and reptiles, whereas only the new ancestral gene was acquired by eutherian ancestor about 90 Myr ago [36], [43], ultimately forming four members of mammalian lineages by lineage-specific duplication. The three platypus IFIT proteins were possibly generated by specie-specific duplication events after its divergence from mammalian lineages around 166 Myr ago, since these proteins are actually co-orthologous to subgroup IFIT5 members of eutherian mammalian lineages.

Interestingly, there is a similar evolutionary history for vertebrate IFN gene family and IFIT gene family. First, IFN genes and IFIT genes are found in the genomes from the jawed vertebrates including elephant shark but not from invertebrates. Second, similar to IFIT gene family, IFN family genes have undergone extensive lineage-specific expansion [27], [28], [29], [45]. Finally, there are varied numbers of IFN genes in different fish species [28], [45], and of IFNα subfamily genes in different mammalian species [46], indicating that IFN gene family has also expanded through species-specific gene duplication.

Subsequent expression analyses of zebrafish IFIT family genes reveal functional association of both IFIT and IFN gene families since generation of jawed vertebrates. In mammals, the biological function of IFIT gene family is tightly related to IFN response, as these genes are directly regulated by IFN signaling, which is triggered by IFN as well as IFN stimuli, such as viral infection, dsRNA and LPS ([20], Diamond, 2013). Previous reports showed that all-*trans* retinoic acid (ATRA) can induce IFIT gene expression and subsequently, ATAR is identified as an effective IFN stimulus [16], [18]. The molecular mechanism underlying mammalian IFIT gene induction is the presence of ISRE motifs in their promoters, which contribute to their transcriptional induction by IFN signaling [25]. It is reported that mammalian IFIT genes can also be induced independently of type I IFN, by IRF proteins (such as IRF1, IRF3, IRF5 and IRF7) or through signals triggered by PRRs (such as TLR3/4, MDA5 and RIG-I) or PARPs (such as dsRNA and LPS) [21]. Although these pathways are largely unclear, it is evident that these signaling factors can simultaneously induce robust IFN response when they are activated [1], [2]. Therefore, the induction of IFIT genes under these conditions seems to be still related to IFN response. In the current study, we found that the ISRE motif is present in either mammalian IFIT genes or non-mammalian vertebrate IFIT genes (Figure 6), suggesting that predilection of IFIT genes for ISRE motifs was determined at least 450 Myr ago since divergence of fish lineages. Several genes do not contain ISRE motifs (Figure 6), possibly due to loss of ISRE motifs during evolution. Further experiments demonstrated that zebrafish IFIT genes, if having ISRE motifs, are upregulated by IFN and poly(I:C), but IFIT5A and IFIT12D without ISRE-containing promoters are not transcriptionally induced under the same conditions (Figure 5). Consistently, IFIT5A promoter does not respond to IFN, poly(I:C) and IRF3/7 (Figure 6 and 7).

Actually, fish IFN can trigger ISRE-containing promoter-driven lucifierase activity [31], and ISRE motif is necessary for induction of fish ISGs by IFN, poly(I:C), IRF3 and IRF7 [32], [33], [34]. Therefore, the data presented here provide insights into coevolution between IFIT gene family and IFN gene family, because both families share a similar evolutionary mechanism and importantly, the ISRE motifs that predispose recognition by IFN signaling appear to have existed in IFIT ancestral gene promoters, which links both family genes to fulfill common biological processes. It seems that neither could exist usefully without the other. One the one hand, IFNs have to induce the expression a set of ISGs, including IFIT genes, to exert biological actions. On the other hand, the IFN-induced expression character of IFIT family genes enables them to associate with IFN response. Functionally, IFIT family genes have recently been characterized as inhibitors of cellular and viral processes, including translation initiation [5], [6], viral replication [7], [22], [47], [48], IFN signaling [49], [50], cell migration and cell proliferation [16], [51], all of which seem to be related to mammalian IFN response. ISRE motif is present not only in IFIT genes but also in the other ISGs, such as fish PKR [50], fish IRF3 [32], fish IFN itself [31], and all mammalian ISGs [1], [2]. The specificity of ISRE motifs to ISGs enables these genes to work cooperatively during IFN response. Simply, the ISGs can be divided into two classes: the first one originated simultaneously with IFN gene family, such as IFIT family, and the second one that is predisposed in the invertebrate genomes and later selected by vertebrate IFN system during evolution, such as IRF gene family [52]. The different status of the second type of genes before and after selection by IFN system is likely ascribed to whether they have acquired ISRE motifs. An example is that fish IRF3 genes possess ISRE-containing promoters contributing to their induction by IFN [32]. However, it does not exclude that some genes without ISRE-containing promoters are involved in IFN response, such as MITA and TBK1 [33]. Interestingly, during evolution mammalian IRF3 genes lose IRSE motifs and thus do not respond to IFN and IFN stimuli [32]. The gains and loss of ISRE motifs result in a rough regulation of IRF3-mediated IFN response in fish and a precise one in mammals [32].

If our hypothesis about coevolution of both family genes is correct, how can IFIT and IFN molecules appear within a relatively short space of time? How can ISRE motifs are selected to link both family molecules? A reasonable explanation is that nature selection does. Obviously, with the occurrence of complicated vertebrates, an intricate regulatory network needs an improved and effective immune system to protect organisms against microbial attacks; therefore, infection disease is a powerful selection force that might directly drive the appearance of both gene families in the vertebrate ancestors and subsequently shape their functional association by generation of ISRE motifs in vertebrate IFIT gene promoters. The creation of functional association may favor hosts to develop an intricate and effective IFN response system during radiation of vertebrates.

## Materials and Methods

### Database Mining and Sequence Analysis

Using human, mouse and crucian carp IFIT protein sequences as baits, TBLASTN search was performed against the genome database from NCBI (http://www.ncbi.nlm.nih.gov/mapview/) and Ensembl (http://www.ensembl.org/info/about/species.html), including ten mammalian species: human (*Homo sapiens,* Build 37.2), chimpanzee (*Pan troglodytes,* Build 3.1), rhesus monkey (*Macaca mulatta,* Build 1.2), mouse (*Mus musculus,* Build 37.2), rat (*Rattus norvegicus,* RGSC v3.4), cattle (*Bos taurus,* 5.2), horse (*Equus caballus,* EquCab2.0), pig (*Sus scrofa,* Sscrofa10), dog (*Canis familiaris,* Build 2.1) and platypus (*Ornithorhynchus anatinus,* Build 1.1), two avian species: chicken (*Gallus gallus,* Build 2.1) and Zebra Finch (*Taeniopygia guttata,* Build 1.1), one reptilian species: green anole (*Anolis carolinensis,* AnoCar2.0), one amphibian species: western clawed frog (*Xenopus tropicalis,* JGI 4.2), and six fish species: zebrafish (*Danio rerio,* Zv9), torafugu (*Takifugu rubripes,* FUGU4), spotted green pufferfish (*Tetraodon nigroviridis,* TETRAODON8), three-spinned stickleback (*Gasterosteus aculeatus,* BROADS1), medaka (*Oryzias latipes*, MEDAKA1) and elephant shark (*Callorhinchus milii*, Eshark 1.4X), three invertebrates: Sea urchins (*Strongylocentrotus purpuratus*), acorn worm (*Saccoglossus kowalevskii*) and lancelet (*Branchiostoma floridae*) (Table 1). The IFIT homologues retrieved by first search were further used as sequence baits for TBLASTN. Zebra Finch IFITA, chimpanzee IFIT1P, rhesus monkey IFIT1P1, and IFIT genes from green anole, western clawed frog, spotted green pufferfish, three-spined stickleback, torafugu, medaka fish, elephant shark were identified by GENSCAN. TPR-containing proteins of sea lamprey (*Petromyzon marinus*) and hagfish (*Eptatretus burger*) was retrieved from EST databases by a strict BLAST search (E-value<10^−6^).

Multiple alignments and phylogenetic tree were performed by Geneious Pro 5.4.6 program. Based on the consensus sequence of TPR motifs [WLF]-X(2)-[LIM]-[GAS]-X(2)-[YLF]-X(8)-[ASE]-X(3)-[FYL]-X(2)-[ASL]-X(4)-[PKE] (http://www.ncbi.nlm.nih.gov/Structure/cdd/cddsrv.cgi?ascbin=8&maxaln=10&seltype=2&uid=29151), a putative TPR motif was identified when more than 4 out of the 8 key sites were conserved. The conservation in the other sites was also considered. The exon/intron structure information was retrieved from NCBI genome databases (http://www.ncbi.nlm.nih.gov/mapview), except that Zebra Finch IFITA, chimpanzee IFIT1P and rhesus monkey IFIT1P1 were analyzed by GENSCAN. For gene synteny analyses, approximate 10-million-base-pare sequences flanking IFIT locus were collected, and then compared by local blast software from NCBI (http://blast.ncbi.nlm.nih.gov/Blast.cgi?CMD=Web&PAGE_TYPE=BlastDocs&DOC_TYPE=Download). 5′-flanking sequences of IFIT gene loci were retrieved from NCBI genome databases and ISRE motifs were identified by software TFSEARCH and Geneious combined with manual inspections based on the consensus sequence (G/A/T)GAAAN(1-2)GAAA(G/C)(A/T/C) [32].

### Cells and Induction

*Carassius auratus* blastulae embryonic cells (CAB) [53], epithelioma papulosum cyprinid cells (EPC) [54], zebrafish liver cells (ZFL, ATCC CRL-2643) and zebrafish embryonic fibroblast cells (ZF4, ATCC CRL-2050) were cultured at 28°C in medium 199 supplemented with 10% fetal calf serum (FCS), 100 U/ml penicillin, and 100 mg/ml streptomycin. The recombinant crucian carp IFN was produced by a prokaryotic expression system according to a previous study [31]. For induction assays, cells were seeded overnight in 6-well plates, and then the cell culture medium was removed followed replacement with FCS-free 199 medium containing different doses of recombinant IFN, poly I:C (Sigma-Aldrich) or with FCS-free 199 medium alone as control according to a previous report [4].

### Plasmids, Transfection and Luciferase Activity Assays

Zebrafish IFIT gene promoter-driven luciferase constructs were generated by insertion of 5′-flanking sequences upstream of the translation start sites of DrIFIT5A (–865/+17), DrIFIT12A (–922/+75), DrIFIT13A (–529/+38), DrIFIT17A (–925/+59), DrIFIT17B (–996/+202), DrIFIT17C (–1091/+94) into *Kpn* I/*Xho* I sites of pGL3-Basic luciferase reporter vector (Promega). Wild type zebrafish IRF3 and IRF7 plasmids were generated by insertion of their whole ORF into the *Xho* I/*Kpn* I sites of pcDNA3.1/myc-His(−) A vector (Invitrogen). Wild type zebrafish IFN1 was cloned into pcDNA3.1/myc-His(−) A vector. The primers are listed in Table S3.

Transfection assays were performed according to our previous reports [31], [32], [33]. Typically, cells were grown overnight in 24-well plates at a cell concentration of about 1×10^5^ cells/well. Cells were transfected with various concentrations of plasmids using Lipofectamine 2000 kit (Invitrogen). For luciferase assays, cells seeded in 24-well plates overnight were cotransfected with various plasmids at a ratio of 1∶10 (pRL-TK, pIFIT-Luc) or 1∶10∶10 (pRL-TK, pIFIT-Luc, pcDNA3.1 or IRF3/IRF7 constructs) using Lipofectamine 2000 (Invitrogen) according to previous reports [4], [32], [33]. If needed, the transfected cells were treated with 20 ng/mL recombinant IFN or transfected again with 4 µg/mL poly(I:C) for indicated times. 48 h post transfection, the treated cells were harvested and lysed according to the Dual-Luciferase Reporter Assay System (Promega). Luciferase activities were detected with a Junior LB9509 Luminometer (Berthold) and were presented as relative light units (RLU) normalized to the amount of Renilla luciferase activities. Each independent experiment was carried out in triplicate.

## Supporting Information

Figure S1 Phylogenetic tree of 107 IFIT family proteins by Geneious Pro 5.4.6 program with maximum likelihood method.
(PDF)

Figure S2 Phylogenetic analyses of 102 vertebrate IFN family proteins by neighbor joining method.
(PDF)

Figure S3 Analysis of promoters of IFIT genes identified in this study.
(DOC)

Table S1 Definition of IFIT genes identified in this study.
(XLS)

Table S2 Identities among IFIT proteins and TPR-containing proteins that were identified in this study.
(XLSX)

Table S3 Primers for expression analysis and luciferase assays of zebrafish IFIT genes.
(DOC)
