# Supplementary figures and images for: Lineage-Specific Expansion of IFIT Gene Family: An Insight into Coevolution with IFN Gene Family
Source: PLoS One. 2013 Jun 20;8(6):e66859. doi: 10.1371/journal.pone.0066859 (PMC3688568; doi:10.1371/journal.pone.0066859)

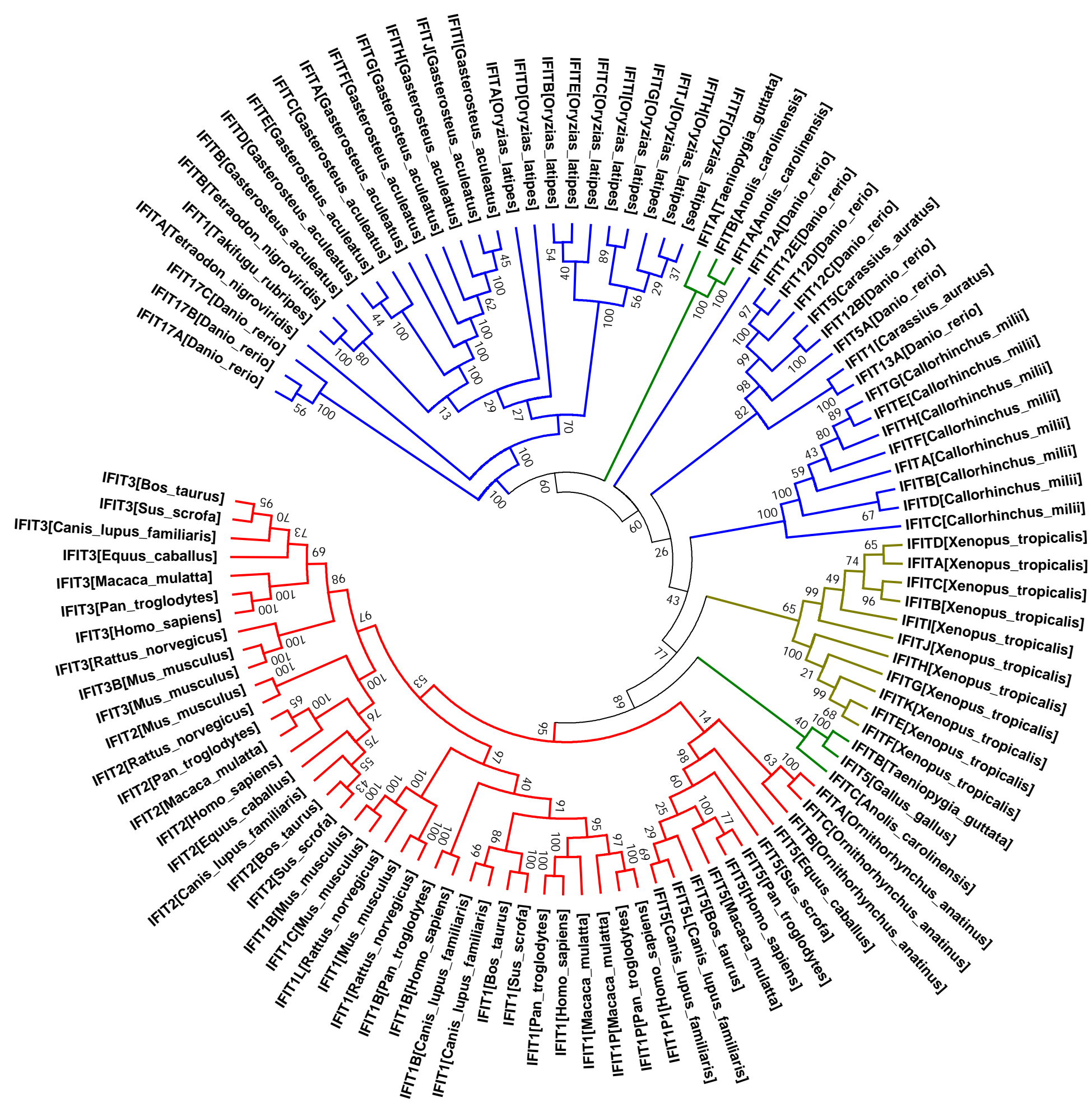

Supplement: Figure S1 — Phylogenetic tree of 107 IFIT family proteins by Geneious Pro 5.4.6 program with maximum likelihood method. (PDF) [file pone.0066859.s001.pdf]

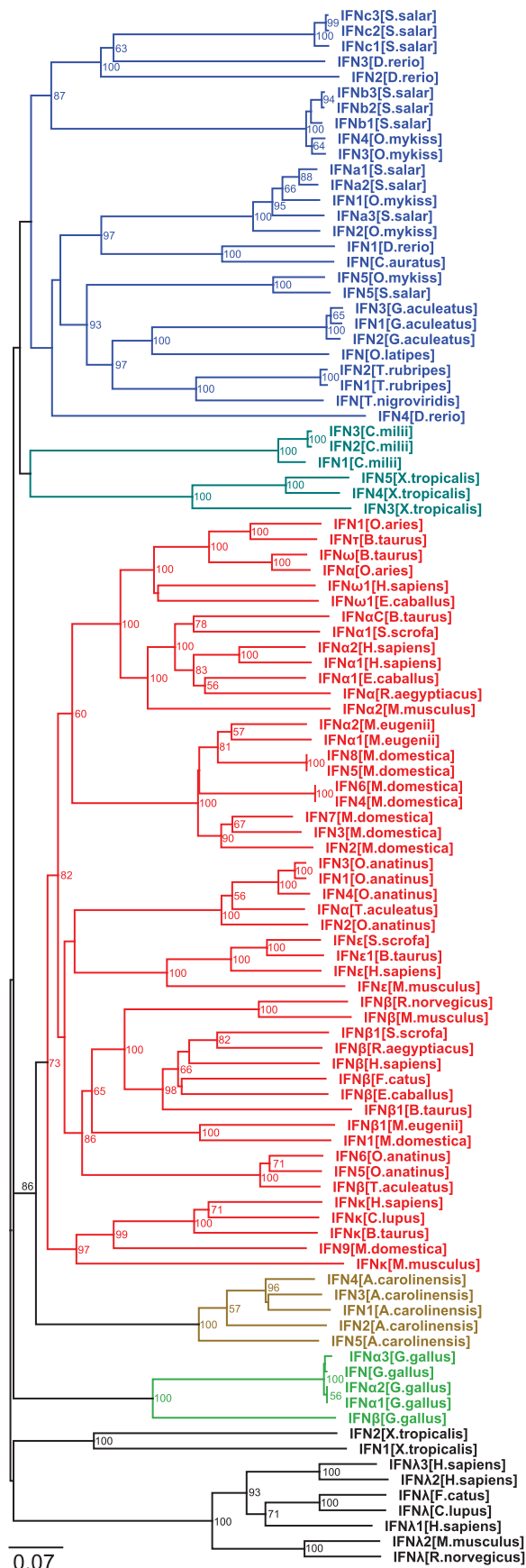

Supplement: Figure S2 — Phylogenetic analyses of 102 vertebrate IFN family proteins by neighbor joining method. (PDF) [file pone.0066859.s002.pdf]
