# Supplementary material for: Lineage-Specific Expansion of IFIT Gene Family: An Insight into Coevolution with IFN Gene Family
Source: PLoS One. 2013 Jun 20;8(6):e66859. doi: 10.1371/journal.pone.0066859 (PMC3688568; doi:10.1371/journal.pone.0066859)
Supplement: Figure S3 — Analysis of promoters of IFIT genes identified in this study. (DOC) [file pone.0066859.s003.doc]

>IFIT5A[Danio_rerio]

CAGGGGGCGATCTAACGGCCGAAACCTTCACTCAAACGTAGGCTTGCGGCACACTTGAAA -933

TTAGGGTATGAAACCCCACTTTGCAGCCTTGAGTCTGCAAGTGGTTTATACGAGCGGTTA -873

GAGTCCGTGAACACCATGCGGGAATAATGAGTTCGAATCCATACTGAAACAATTATTTTG -813

AAATGCGTCAATAGCAGTTGGTAATGCTTTGTTATTGTATCGTTTTGTGTCAACATTGCT -753

ATGACATACGAATAAACACCTTGTAAATAAATTTGTATTGTTTTGTTCATAAAACAGGGT -693

TGTTGCTAGATCAGATACAGTTGTGGCCAGAAGTTAACAATAATTATTTATATTATTAAT -633

TAAATTTCCCATTGTATTTTATTAACGTCTATCCAAAACCTAAACCCATCACAGTACTGT -573

AAAAATATGAATTATTGTTTTACAGTGTTACAAAAATGATGCTAAATCGATGGCATATCC -513

GTAGCTGTATCCTATCTAGACTACACCATAAAACAGTAACATGATTAAAATATATAAAAT -453

CATGATTTTGTAGTATTTGGACTAGTCGGGATTCGAACCCAATATAAATTTGTGTTGTGC -393

ACAAGCACGATCCACTAAGCCATACAAAACACAGTTACATCGTTGATCATGCCAGTCAAA -333

TGCAGATTCTCCGGGTCATGAAACGCCTCTGAAATGATCAATGCTTTGAATCGCTTTAGT -273

CACGTGACCAGGTGTTTCGAAACAATTTAGTCACGTGACCTGGGTGTTTCGGATCATGCT -213

TCGGTGCAGTGTTTCGAAACACTTGCGCTTCGGGATCTCGACACTGTGTCGAAACGTCAG -153

TTTCACGTCAGCCATCCTTACCTGAGGCGAAGCTTAGGTTTTGTTTTTAAACTCTTCCTG -93

TTCACCTTTCTGAGCTCGGGACATTTAAAACGTATTTTTTAACTGCAAAAAACTACTTCA -33

GGATCATTTGATTCTTGGTGAAGAGATTCAAAATGAG +5

>IFIT12A[Danio_rerio]

TTCATACTGAAACTGTTATTCATTTATTTTGGGAGTGTAATTATGCTGTACAATTTTGGA -1000

AACATATTGGATTATTTATTAATGATAATATACAACAAGATGTTTCTGTTACTTTTAAAG -940

AAATTGTTTTTGGTTATTATGAATCTGCCCCTACTAAGAGAAATGCTTGTTTTGTTATAA -880

ATTTAATTTATTTTCTTTGTAAATTCCATATCCACAAATGCACATTTACTAATACTAAAC -820

CATATTTTCCTGTGTTTTTGCAAGAATTTAGTCATTACATACAATTAATTTCATTATCTA -760

AAAATAAGAAAGCAAACAGAACTATTTCATTTTGTAATGACTTTAATTTATGTACTGTTC -700

TTGATTCTTAAATGTCTTACCCTCAAGTTTATATCATGTTCTTTATTTTTATTTTATTTT -640

TCTGTCTTTCTCTCTCCTATCTCTTTTCATTGACAATGTAATTCATATTCTGCATTGTAA -580

ACAAATATTGTTACAAACTTGTCAACTGTTTTTTTGCAAAAAAAAAAAAAATAAGGTTCG -520

CTTTGAATGTAAATTGAGAACAACAATAAATGTGATACTCTTCCGTTCCCAGATCTTGAG -460

TAGACTTTCAGGTGAGGATACCAACTGTTATGATAAATGAGGAACAGAGTTCCTACGGGA -400

GAGGGGTCTGGATGCAGACCTGGTGCAGTGCAGTGTAAAATGGGCTTACCTAACCCCACC -340

GCTAACCCTACCCCTCACAGTGTCACTCGCTCCATTTGAGTGCATTGTGTATTGCATCGC -280

TGAGTTATGCACTCTCAGATTGTCTTGCATCGTGGCTGCGACCATTGTGCTGCCGCTCCC -220

TATGTCACAAGAATTAAATTATCATTCCAAACTCCAAAGGCATTATTTAAATGCTTAAAT -160

ACTTCAAGTGAGGAATTCTCTGTGATAATTAGGCTCTTTTAAAAGGAGCATGAAACGCTT -100

GTGAACTAAAGTGGAAATTGGGTCGAGGCAGACTCATTTCTTAACACTAACAGCCCATAC -40

GAAAACGAAACTTATCCTTACTTTCGCTTTTGTGACATCGAATCTTTCCCTACACAGCT +20

>IFIT12B[Danio_rerio]

CTGTTTGTTGCAGTAAATGATAATGACATATATTACATTAATAATATGTTATATAATATA -1008

ACATATAATGTAATTATAATGACAAAGAATAAGGTTTTAAGATGCAGTACTATATTTACA -948

TTGAAAATGATAAATACTGAAAATATATATTTTTTGCATCTGAACTGTATTCAGTGAAAT -888

ATATATTCATATTTGTAAATTCAGTTTAATATTTGTATTGTCTTTAGGCCAAAAAGACAA -828

ATAAATAGGCAGACCGTGTAGACTGACATGACTTGGACTAAACAATTTTACTAACAAATA -768

TTAAGCAAAATTTGAGACAGCCTTAAACTCTCTCTCTCTCTCTCTCTCTCTCTCTCTCTC -708

TCTCTCTCTCTCTCTCAGTCTCCCTTCAGCCAAGGTTTCAGTTTCCGTTTTGCGTCTGAC -648

CTTCCCATCTCTAGGAGAGAGCGTTAAAGCCTGCTTCTCTTCGGAATCAAACGGGTTCTG -588

CATCATACAGCAAAGCTTACAGAGGTCCAAGAACAGGTGAGCACGCCTATATTTGCTATG -528

TTTTTAAAAGCTTAATAGCTTCTGCAATACTATTTACAAAGACTGTAAAAGATCAAGTTT -468

GAAATGGATGTGGGATAAATTTTACTTAAATGTATGCTGACCAAAATAGATATATTTACA -408

TACCATTTTAATTGCATATATATATATATATACACACACACACACACACACAGTTGAATT -348

ATTACCCCCCCTTTATTTATTTATTTATTTATTTTAATATTTCCTAAATTATGTTTAACA -288

GTGCAAAGAAATGTTCACAGTATGTCTGATAGTATTTTTTCTTGTAAAATATCTTGTAAA -228

ATATTCTTTACTGTCTTCATGGCAAAGATAAAATAAATCAGTTATTAGAAATGAGTAAAA -168

CTATTATGTTTAGAAATGTTTAAAAAAGTCTTCTCTCCATTAAACAGAAATTGTGGAAAA -108

AATAAACAGGGGGCTGATAATTCAGGAAGGCTAATAATTCTGACTTCAACTATATATACA -48

ACATATATTTCATATTATATATATAAAAAAACTTCATTCAATTTTAGGATGACTTCAGAT +13

>IFIT12C[Danio_rerio]

AGCATTTAAACTAGCAGGCTGCAGGTCCTTAATGAAAAAGCAATACATTACAATGGCAAA -1118

TTTATGCCTGCTTCCCAAAAACTTCAGAAAATGCATTCCAAAATAAATTGATCAGAAACT -1058

TTTTTCAGTTTAAACTTGTGTATTATTAATACAGACAGTTAATTATATGATAATTTTACT -998

AAAAGCCCACAAAAAAAGTCTCTATAAGTTTTGATCCGATAATTATTAATGATACATTTT -938

GTTGTGGCATTCATTTGTACATCATAATACAATGATTAATTGAAGTCAGCATTCTGAGGC -878

ACTTAAATTCTTGCCCGACCCAGTTTTACAGATAGCTGAATTGTTGTAATGAAAAAGCCT -818

TAATCACACGACTTTGTAAAAATGTTTTCATACTTTAAAACATTCATTCAATTTCTTTTC -758

GGCTTAGTCCCTATATTAATCTGGGGTTGCCACCAAGGAATGAACCGCCAACTTATCCAG -698

CGTGTTTTATGCAGCGGATACCATCCTATTTCAAAACAAAAACTTTTTACAAATATTTTA -638

AAAGTGTATTAGACAAACCACACTCTAATGAACAGACCCAGCTGAGTCAAACTAACAAAC -578

AAAAAAGAACTGATAAAATAAGACTTCTTATTAGGCTACTTCTCATTGAAAGTGATTGTT -518

GTATCAGCATTGAGTAAGTTAATCATGATTATGGAGAGTAAAGCTATTACCTGTGCATGT -458

GCTTTATAAGTATTAGTAGATTTCCATACTAATAGACATAAATCTAGTAGGCTAATGTGC -398

TCACAATAAAGTCACAGTTTGTAGCATGCAGTAAGCATAACAAAGCAAAAAATGGCTGTA -338

TTATTTATTAATTTATTTGGATATAAAAAAATAAATGTATTTTGCTAGGTAAAAATTTTA -278

GATGCATTTTTATTTACTGATCTGTCCTCGTATGATCTAAATGTTACTTCATATTTGAGT -218

AAAATAGATCTATAAAATACTGTACACTATTAGACATATTCTTAAAATAGCCTATTATTT -158

AGGGGGCTGAAGGCAAATTCGAATTAATTTATACAATTTAAGTATATTAGATCTATAATT -98

AGGTATTTAGAGTGTCATGAGTGGGCCTGTTACGAAAGCTAGTTTGCCTTTTTGCATCTC -38

AAACAGGAAACTGAATGGGCATGGTGAGTTTGGTGACGTATTCAGGAACTGAAA +17

>IFIT12D[Danio_rerio]

TGACATTTCTTTGTGGAGTTTGCATGTTCTGTGTTCGCGTGGGTTTCCTTACTTCGGGTG -977

CTCTGGTTTCCCCCACAGTCCAAAGACATACGGTACAGGTGAATTTGGTAGGCTAAATTG -917

TCCATAGTGTATGAGTGTGTATGGATGTTTCCCAGAGATGGGTTACAGCTGGAGGGGTAT -857

CAGCTGCGTAAAACGTGCTGGATAAGTTAACGGTTCATCAGTTGGGAGTTGAGCTAGAGT -797

AGGGAGCTGGAGGCCAAGTGAAGGGGGTGGTAAAAGGTTGGTCAGTGTGTGGTAGAGAAG -737

GGTCATTGTCCAGGGGAATGCCCATTTGTTGTAGTGATGTTGAGGCTGGGGTGTTTCTGG -677

TTTCTTGAAGGCGTGATCTAGATAATTTTTGGCTGTGGGCAATGCTTTTCCTTGTGGCTT -617

TTTTTGGGTGGTCCAGGTTGAGATTTTTGTTGTAGAGTTGGTGATTTGAGGATAGGCAGA -557

GGTGGGTAGAGTATCCAAAATCTATACTCAAGTAAAAGTACAAGTACTTGCAGAAAGTTA -497

TACTCAAGTAAGAGTAAAAATCTTAGAAGTAAATGTAACAAAGTAAATGGGTCACACTTT -437

ACATTAAGGTTCATTAGTTAATGTTAATTGATGCATTTACTAACATGAACAAACAATGAA -377

CAATACATTTACTATTGTATTTGTTCATGTTAGTAATCGTTAATGAAAATACAGTAGTTC -317

ATTGTTAGTTCATGTTAACTCATGGTGCATTAACTAATGTTAACAAGCATGCACTTGGAT -257

GTTAATAATGCATTAGTAAATCTTCAATTATGATTAATAAATGCTGTACATGTGATGTTC -197

ATGATTAGTTCATGTTAGTAAATGCATTAACTAATGAACCTTATTGTAAAGTGTTACCAG -137

TAAATGTAATTGTTACTACCCATCTCTGACGATCTTGTAGTGTCCATATGAAGCCCTTAC -77

TCACCTGTCAAGATATGAGGCACTATATGTAGTGTAGTACAGTTTCAGTAATTCTGCTTT -17

GTTGCAGTGTAAAGGTATGGGGGTTT +10

>IFIT12E[Danio_rerio]

AATTCATACAGTTAACACCAATTCCAAATGCTTGAACGTCTTATACACACAAGCTCAACC -897

AACGCCAAAACAATGTCATTTTACAGTCAAATTTACAAATGCTTTCAAACTGTATGTCAG -837

AACACATAACATCGTCTTCTAAACAGAACTTAGGCAAAAGTCATAGTGAACAGTTGTTCA -777

TATTGTGAACTGAAACAAATGTATTCCCTGTATTTTATTCCATTGCCAATGAGAAACAGC -717

TTATTGCAGTTATGCAAATATATCAATTACAGCAGATTCCCATCTAGGAACCACACATTT -657

TTCCAATCACTTGATCATATGTTCTAAGAGAGGAGTCTTTGGGCTGATCTTGAGTGGAAA -597

AGGAACAACAAAAGAATTCTTCCCATAAAGCCTAGCCAGAGAAGATATCAAGTGTGATGT -537

GGATGAGAACATGTGGCCAAATGCAGATAACCAGGCAAAATAGATGACTTTTTTTATTAT -477

TATAATATTATTATTACTTTTTTATTATAAATGATTTATACATGATCTCTGCTGGGGGGG -417

GGGGGGGCAGCATTGAAAGAAGAAAAGTGGATGCCATCTCACAATGGGAAGAACCTAAGA -357

CGTTAAAGGAGTTGCAGAGGTTTCTAGGATTCGCCAACTTTTACCTAAGGTTTATTAAGA -297

ACTATAGTATTCTCTCTGCACCTTTAACCTCCCTCCTGAAAGGCTGGATGGAAAGCTGGA -237

TGTTGTGCTAACTGTGTTAGGAGTTTAGGAAACTGTTAAATGTACTGAAAAAACTATCAT -177

AGAAATTGTAAAAAAAAAAAAAAAAAAAAAATCTAAAAGAATGACACAGCAGTTTAGACA -117

AAACAGCATCCTGTAAATCGACACTTGACTCATCTGAGGGGCGTGATTTAGCCTTACAAT -57

TACAGGAAATGAAAAACGAAAGTGCTGGGTGGTAGTCCACGAGTGATACTTTGTGCTTTT +4

GTTTTTCACAAAAGAAAAAGGATGTCAGAGATGAG +39

>IFIT13A[Danio_rerio]

ACGACCGAACGATCATGCGTATGTTTGGCCGACCATATACCGATGTTAGAATTAAAGGGG -916

CCGACGTGTTGACCTTCGGCCGACGTCAGCCCAACGTATGTGTGCTGTCTGGGATAGCAC -856

TAATTAAAAAAAAATAACAACACCATTTTAAAAATGGTGAAAATCTAAGAGGGCAAGAAA -796

ACTCTCTATATATTCTATAAAACAAATCGCACAATTATAGGCCTAAGTTATTATTTTGGA -736

GGTTATTTTCTCAAGCTCTGATGGTGCTTTTTTATTGAATACAAAAATAGCATCACTGCA -676

TTGAAGAGAAAACACAATAACAAAAGATTAGGAAAGAGAAGAAGAAAAAAATACTTTACA -616

AAGCCCCGATCAATCAGAAAAAAGATTATCATAAGCATTAAAAAACGTATTTTTCTTATT -556

ACTATAAACTTAAGAGATTCGCTCTTATGTAGCCTTCAGACGTTATTTGGCCACAAGATG -496

GCGTCATTACTCTTCAATTTTGGGTCATTCGTCCCGTACAAAAACATGTGTTGACATGTC -436

AGCACCAGTTAGCACATACCGCCTATAAAGTAATTATTGACGGGTTTCCTTATCATTTTC -376

TTGTACTTTACTATCTATAATTGATATTTGAGGTGAAAACTTAATGTAATCAACATAGAA -316

AACAATCTGTAGCATAGCCTACATTTTATTTTTTTTCTTTATCTCTCCTAATCTTTTAAA -256

GTTGTTTTTTTTATTTTGTTTTTATTAGTATTAGCTATTATTTTATGCATAAAGTGAAGC -196

ATTACATTCTAGCGTTTTTAGTTTAGTTTAGTTTTGTTTTGTTTTGTTTTGTTTTTTTTA -136

TCGTACCCTATCATGCAACCGGTAGTTTCGTTTATTCTTAATCAGAGCATACGTGAGAAA -76

TGAAACTTAACTTGTAATGTCCCATGGGACGGTTACAGCGACACACTATAAAAACCAAAC -16

AGAGAGACCGCATTTATCCAGACGTTTTGATTAGAG +21

>IFIT17A[Danio_rerio]

AGTAAATAAAAAGACAATAAAGCCATAAACATTTTTCAAATTGTGGTACATTCCAGCAAG -984

TCAGTAAGACAGGAGAGTGCTCCCTCTTCGAATGTTATCACACAGTTAAATGGGTGTTAT -924

TGGTGGTTATTAGCTGATAGATATCGGCCAGTAGGGACCTTTGCACTTACTGATATGCTC -864

AGAAGGCTGATATCATCCATCCACATGGTTAATCAGCTATACTAATAAACAATCTGAGAT -804

CTGTTTTTACATTGCTGTGATGGTTGGGTTTAGGATAGGGGTAGACATTAATATAATACA -744

ATTAATAGGAAATGAAATAAATAATATGAATAATTCTTGTAAACTTTCAGCCAAAGCCGT -684

ATGTGACCTATAACTGATTAACCATGTGGATGGATGATAACAGCCTTCAACGCCCATTCA -624

ATCGAGTGATGAAAGTGTTGGTATAATGCTGGTGTATTCTTGTCAAATTAAATCTGATTC -564

AGTTCAGTTGAATATAAATGTTGAGTTTAATTCAGATCAGTTTTATTGAAATGTCACTGC -504

CGATCCACCGGGTAAGCATTAAAACTGTAATCCTGTGTTAAAATGTCAGAATAAGTAAAA -444

AGTAGGTGTTTACAAAGATACATCTGTAAGTGCAGAGGAATTTCACTTGAAATCATAAAA -384

AACTTTAGAAGATAAAATCTATTAGATAAAAGGCTTTAAATTCAACCATTTTCTGTCAGA -324

TTTCACCTAATTGGTCTTGCACAGGCCTAGCAAAATGATATTGTATCAGCTTTAATTCAC -264

ATTTCTCATATTCAGCTTAATGGCGTTTAGATCATCACCACCCTGCCCTGTAATGCTAAC -204

AGTGCATGTCATCAATAGGATTTTCCTGGTTCATATTGCGTATAGTTATCCTGAAATGCT -144

CTTTGGACTCTTGTTGATTTACACAAAAACACTACCATGTGAAGAACAGAAACTTATGAG -84

GAGGATGTTGAGCTCATGTTTTATAGAGATAAGATTCCTTTCTCCATTATAAATACAGAC -24

ATCAGCAGCTCCAACAGTGTTCTCTGCTGGAGAAAACACATCT +20

>IFIT17B[Danio_rerio]

AAAGTGGGTATCAGACTAGACAAAAAGCACTGAATTAAATTCCATTGTTCTGCGACATGT -1060

TTTTAAAAATATGAAAATTCATTTTACCACAGTATTTTCAATGGCGCTTTGGTGTTTACT -1000

TGCTGCTTCTGATGGCACTTGTGTTTTAAACTGTCTAACTTGAAACTTTCTCATTTTACA -940

CTTGAACAAGTAAATAAATGCTTAAGTCTACTTAACTGATTTTATTTTAATGACATTACA -880

ACATTGATGCTGTAAAAGGAACCATATGAAAATTAAAAATTATCACAATAACTTTTCACC -820

GGACGTTTATCGGTGGCTGAAAAACACTCGCTGTGCATATAGCCCGATTCACCTTCATTC -760

ACCCTATGCATTGAAGACATTTTTCCAAACAATGCTTTCCATTCATCTCCAATTTGCAGA -700

ATTACAGTATAGTTGATAAGTTATCTCTATTCAATGACATGACAGACTTTGAAACCATTA -640

AAATTCCCCTTGCGTTGCCAGTTATAAAAACACAAGTTAAATGAAACTTAATGAAATGAA -580

ACTTAAAGGCTGTATGTCCTCATTTCCAGCTTCTAAAAATATTTTCTTTATTTCTGTCTA -520

TATAAATCAAGTTTAGAATTTACATTAACTGATGCTAACAAAATTAATATCACAAATGTA -460

TTGTTCGTTGTTTACATTATATTTGATATATTGTAAGCAGATGACTTCAACAGTCTGAAC -400

TGCCAGACAGAAATACAACAGGTCTAGTCTTCACAATTGCATCAACGTTTCTTGCAAATA -340

AAATTTCTGTTTTATTGTCATGAAATATGAGTTTACTGATTAAAAATAATGACCACACTA -280

ATTTGCATTAATGCTAGAAACAACATTGTGTGTGCAAAAATAATAAATTAATGCAACACA -220

TCCAAGAAATTTGCACCAAAAGGAGTAGAAAAAAAAACATTGAACTACAAAGATGTTGTG -160

CTCTTAAACTGGCATCTTAAGAGTTTCTGCTTGACATCTTAATCACTTTCGGTAAACTAC -100

ACTTGAAGGAGAAATGAAACCAAGAGTATAATCTTGAGCTGATGTGTTTAATGACTTTGT -40

TCAGGTAGATAAGACATGAGCTTGATCATTTAAATACAGCATGATTTATCAGTACC +17

>IFIT17C[Danio_rerio]

GTGAAATTTGCTTGAATGTTACAGCGATTGGATATTCTTTAAGACGAAGATGACTAAAGG -1174

TAAGATGCTTGGTTATTTATACTGAGTTAACCTGCGGTCACACTGGGCTTTTCCTCCCAT -1114

AGACTTCCATTCATACGCACACGAATACGTCAGACCGGAAATGCAGGGTCATGCTGTGCA -1054

ATGTTCAAGCTTGGTGAACTCTGACCTGCGAAACCGCGTCACTAGACTCCGTGAGACCAA -994

TCGAGGATCAAAACATGACCTCTCTGAACAGAAATTTAAAATATAGAGCAATCGCTTACT -934

TTGTAAATGTCTAATCATCTTGTTTAGCCCGCCCCTTTTCGCAGAGAGGTTAGACAGAAT -874

TTCGCAAACTCAAACTCTGGTGTGACCGCAGCTTTAGGACTTGTCTGGTCGGTGGTTCGT -814

ACATAAGCTTGACTCGGGGCCAGCCGTGTCAATCATAAGCACGTGCTACTGTCGAAATTT -754

GATTATAAATAAACTATACATAGGCTACAGATTTTAGTTTAAAATTTGAACAGATTTCTA -694

TTTTTTTCTAATGATGTAATGAATTTGTATTTAATCTAAATCTTTAGGATGTATTTTGGT -634

ACAGCAAAATGTGTGGTTATTCTAACCATCCGACAAGCTAATCTAGCAAAAAATAAATCT -574

TAAAATGTCACTAAAAGCAGTACTCAAATTTCATAATTAAATAGAAAATGAGGCCACACG -514

GAAAGAACCTATATGAACATATGTGTTTGGTATAGGTTTGATGTAGGTTCATATATGTTT -454

ATATAGGTTATTTTCCGTAACTGCAAAAAGTTACATTTTTAAAGAAAAAAGAACCACTCC -394

TTTAAACAGACTGTCTACAGGTCTGCTACAATACAGCTTTGTTAACCACAAGAGACCATA -334

GATAGGATTTACCAAAGCATTTGAAAAGAATGCCTCTGAAATTTATACTCTGCAGACAGT -274

AACTTTTGTTCAGTATTTTGAATCAAATAGCGCACAAAGGGGGTTAAACAGATTGTAATT -214

ATTATGTTTAGATGGGCTAGAACTAAATGACATTGCAAAATATGTTTAAAAATACATTTC -154

TATGAGTGTGACAATTGTTTTCTTTCTCATTCCTGATAAAGCCTGCTGTTCGCGGGGTGG -94

CTTGTATCCATCAGAATAACAGTAACTGACGTCATTCCGGGAAAGCGAAACCAAAAGCAG -34

AAACAGGACTGAATGAAGAATCGAAACTTAGCCTACAACTTTATTACCCGTG +19

>IFIT1[Homo_sapiens]

GAGAGGGGGTTTCACCATGTTGGCCACGATGGTCTCCATCTCCTGACCTTGTCATCCGCC -792

CACCTTGGCCTCCCAAAGTGCTGGGACTACAGGCATGAGCCACCGCACCCAGCCAAGAAT -732

CATTATTTTTAACTTGATGACTGAAAATAATAATAATAATAGTTACCACTTATTTGCATG -672

CTTCTATGTGCCAGGTAGTTGCTAACTATTTAAACTCAAATTCCATGAACTGTAGTGGAG -612

GTTGTACTGGAATTTGATTCAGAATGACAGTGTCCATGATGGAGCAATAGAGGGCTCTCT -552

ATTTCAAACCATACCTCCTTGCTTTTACCTCCTGCCTAAGTCATCAGGGGTTAGAAGGCT -492

TTCTAGGTATTGGTCTCTTTCCTTCATTCCTAAACCAGATTGGTTGCTTATTTCCGTCAA -432

GCTGAAACCAAAAGTAAGCAACCAAAAAGCAACCAGCAACCAAAAGCCTTGTTACTCAAT -372

TAATTAAGAGTAGATTTTTATATTTGATAGTAGGTTCCTTCTAAATATAGAAACTGAAAA -312

TAGAGCTATCTCCTTCAATTCTCCTTTTTCTGTGTATTCATCCAGAATCCAGCCACCAAC -252

TGCCACAATAGGCAGCAATGGACTGATGTTCTTTAGGGAGGACGTGAATCTCGTTCCAAA -192

TGCTGGCCAGTCATTGGGTTTCTGCAGCACTAGAAACATCTATGGTTGCAGGTCTGCAGT -132

TTATCTGTTTTAAAATAGAAACAAAGTTTCATTCCCCACCCCCCCCCGTCAGCAGGAATT -72

CCGCTAGCTTTAGTTTCACTTTCC CCTTTCGGTTTCCCTAGGTTTCCAACTTGCAAGGAC -12

ACACCCACAGCTTACACCATTGGCTGCTGTTTAGCTCCCTTATATAACACTGTCTTGGGG +49

>IFIT1B[Homo_sapiens]

CTTTTTCTCTGCTAATTGTGTGGGCAATTACATCAGCCTTGGGATCATATAAATTTTTCA -873

TTAAACATGTGAACATTGTTTGATTGACTCTCTGATGGTTGTTGCTTTGTAACAGATAAT -813

CTTCAAAATAGTAGAAACAGAAGAAACATAAGGACAACTCAGAAGAACTATGTGAATTTG -753

GATTTAGATTTTTAACCCCCATGACCCCATCACATTCTCTGACTACATTGCATATGTTTC -693

TCTACTGACCAGCTCAAACATTGTTGTTTGAACCACTAATGGTGAACAGTAAAGATCATC -633

AAAACAGTCAATATGCTTGAATAGGCTCCACTATAGCCAGGGTTGGCAGTAATGCAAATG -573

CTGCTGGAGGATGTAACCAGTATGGGACCCTTCTTATAGACCAATATCTCAAAATTGAGG -513

AGTTGTTTTGCCTAACACAGCCCCACAGAAGAAGACCTAACCATCTGCAACCTCATTCTT -453

CCACAAATTGACAATGACTTTCTACAGTATTCTTTTAGGCACAAACCACTGACCCTGGTC -393

TAAGGAGCTTCCATTGTAGACGTCCCTGGAACCACCTGGCGGGATACATGTGATAGTAAC -333

AAGTTTGACAACAGAGTCTGACATGATTCACAAAAATATACGTTCCTGTCTGCCACTGGT -273

TTTTCCACAACACTGAATGTCATCATAAAGTCCAGTTACCCTCTATCTCCTACACCACAT -213

TGACTCCAGCAAGGGAATTTGCGTATCAAAACTGCTTCTCACACACCCATCCTCTTAGCT -153

GCCAAAACTCAGCATGTATTTGTAATCAAAGCAGAGACAGCCCTATTCCTCTGTATCATT -93

TTCACCCTGCCAATTCACTAGGCATCAAGGGTGTAAAAAACTGCCTGGATATACCTCCCA -33

TATATAAGCACCATGCTTATCTGAGAAGCCCTAGAACTCTGTGGATGAACCTTGAAGGAG +28

CCTCCAAGCCTGAACCAAAGCACTACAGATCACCTGCTATCTTCATAGCACCATGAG +85

>IFIT2[Homo_sapiens]

TGTATTATTCCCAATTTACAAAACCGAAGCAGAGACATTCAATAATTTTCCCCAGAGTTT -873

CATAGCATAAAGGTGGAAGCTGATTTCAAATCCAGATAGACAGACATCACTGCCCATGCT -813

TCTAATCATGTATTCATTTAGTCAACAACTACTTTTGGAATGCTTGCCATACCATGCAAT -753

GTGCAGGTTGTTTTGCAGGGACTGGAGATTCAGCAATGATTCAGACTAAAATCCCCTGCC -693

CTGTCGAGGCTAAATAACTGCTCCTTACAGTGGCCTGTGGATCATATTGGTATAAGGCAG -633

AGCAAAGATGGGTTAATATGGTAATCACAAGGCAGGGAGCTTTGTAAATGTGTCCTCCTA -573

GGAGTTGTGCTCCCTTGATGCCATCAGTACATTCCACAAAGAGGAAGTTCTTCTAACTCA -513

CTACGCAGCTGGGCTTGGCTCTTACTCAAGAGCATTTTGGGGTGAAAATATCTAAGTTTA -453

CCAGCCCTTGTGCAAATCTGAGATGAAGGACTACATTAAAAGTAAAATTCACCTTAGCAG -393

GAAGTGGGGTTTGCTATTCTGTGTATATCTTTAACAGTATCTGCTGAAAAGAACCCTTTT -333

GTTAAATAATTGTATTACAAGTCCCCAACTTAATCTTTTCGAATATGAAATAAGAGAGGG -273

ACAGTGCACAAGAGCAATGTCCCCAGACCCATCTTTAAGTGAAGCACCAGGCCGATGAAA -213

CATCCCTCTCTGCTGCCTTCTTTCTCTGATCACAACTCAGCTCCGGAGGAAAAAGAGTCC -153

TCTAAAGTATAATAAAAAGAAAAAAAGAAAAAGAGTCCTGCCAATTTCACTTTCT AGTTT -93

CACTTTCCCTTTTGTAACGTCAGCTGAAGGGAAACAAACAAAAAGGAACCAGAGGCCACT -33

TGTATATATAGGTCTCTTCAGCATTTATTGGTGGCAGAAGAGGAAGATTTCTGAAGAGTG +28

>IFIT3[Homo_sapiens]

AGGCAGATTCAAAGATTTTCTGATTGACAATTTGTTGAAAGAGTTATTATCAATAGAAAG -893

GAATGTTTGGAGACATCCAATAGAAATTCAATAGACAATCATTAGAAAGGAATGTCTGGG -833

TCGTGGAGACCAAGGTTTTATTATGCAGATGAAGCCTCCAAGTAGCAGGCTTCAGAGAGA -773

ATAGGTTGTAAATGTTTCTTAATACACTTAAAAAGAGCATGTTCTGGCTGGGTGCAGTGG -713

CTCATGCCTGTAATCCCAGCACTTTGGGAGGCCAAGGTGGTTGGATCACCTGCTATCAGG -653

AGTTCAAGACCAGCCTGACCAACATGGCGAAACCTCGTCTCTACTAAAAATACAAATATT -593

AGCCGGGCATGGTGGTGGGTGCCTGTAATCCCAGCTACTCGGGAGGCTGAGGCAAGAGAA -533

TCACTTGAACCCAGGAGGCAGAGGTTGCAGTGAGACGCAATCATGCCACTGCATTCCAGC -473

CTGGGCAACAGAGTAAGACTCTGTCTCAAAAAAAAACAAAAACAAAAACAAAAAAAGAGT -413

GTGTTCTATTAGTAATTCCAAAAGGAAAAAGGATGTAATGAGGCATATCCAGCTTCCCCT -353

TCACATCAGTTTTCAGGTTAACTTTGGAATGCCCTTGGCCAAAAGCAGGGGTCCAGTCAC -293

ATGGTTGGTGAGTATGGCCTTAGAATTTAATTTTTTGGTTTACATTAGGAATGCTTCCAC -233

ATTCTCTCAAACAAATTTTAGTCACAGTTTTGAATAACGTACTCTGGCTCAGCCATTGCA -173

GGTCTCAAGCCGTTAGGTTTCATTTTCCTCCTCCCAACGATTTTAAATTAGTTTCACTTT -113

CCAGTTTCCTCTTCCTTCCCCTAAAAGCAATTACTCAAAAACGGAGAAAACATCAGCTGA -53

TGCGTGCCCTACTCTCCCACCCCTTTATATAGTTCCTTCAGTATTTACTTGAGGCAGACA +8

>IFIT5[Homo_sapiens]

TCCAACCTGAGTGACAGAGTGAGACCCTGTCTCAAAAAAAAAAAAAAAACCAAAATCAAT -889

GCAAAAGATGAATTATGGACAGGATATTGAGGTTTTGAATACAGACATTAACTCAATAAG -829

GGTAAGGCCCGTGTCTGTTTTCTTTATTGTTATATTGACAGTACTTTGAACAAAGTCTGG -769

CTCTTACAGTTGTTTGTCAATATTTGCTGAATGAATTCAAAGGGGACAAAATGTTTAAGG -709

TATGGGTGTTTGACATTTTCTGCCTAGATCCCCAATACCACTCTTGTCTTTATTGGGTCC -649

TTATTACAAAGCACCATTGGCGAAGGTATGGAAGAGAAGCAGTCTCCTACAATTGTGGGT -589

AAGAGTGGATTTTTAAAGCTATTTGGCAATATCCGTCAAAACTTAAAATGTATATAGATT -529

TTAGCTCCAAATTTCTCAGTTTACACCCAACAGAAATAACAGCATAGATGTATAGCATTG -469

CAGTGTTGCTTGTTGAATCAAAAGACTGGAGTGGAAGCAGGGACTTAAGTTTCCGCCTGT -409

AATCCCAGCACCTTGGGAGGCAGAGGCGGGCGCATCCCTTGAGATCACGACTGGGTGACA -349

CAGTGAGACGCTATCTCAAACAAACAAACAAACAAACAAACAGTTACACGGGCAGCATGC -289

GAACTCAGCGTACTCAGGGAGGCAGATAAAAGCCGAGACCAAAGTGAAGGAGCCCAGCTG -229

CAAATTCAACTCCGAAAGACCCTGGACTCCGCAAACGTCTGGCCGCGGCTCTTGAGCTCT -169

TCTATTAAGTTTCGGTTTCTGAAGCTTACGTTTAGTTTCGATTTCTTAAGTTTCAGTTTC -109

TGAGCGCTCGGCATCTGATTCAATCTCCAGTTTCCTGTTCTTGCTGGGGCTGGGGTCTCT -49

CCTTTAACAAAGACACGCCGCGCGGCCGAGTCCAGGGGCTGCAGAGGCCTGGCGCGCGCA +12

>IFIT1[Pan_troglodytes]

CTCGCTCTGTCGCCAGACTGGAGTACAGTGGTGCGATCTCGGCTCACTGCAACCTCTGCC -934

TCCCAGGTTCAAGCAATTCCCCTGCCTCAGCCTCTCGAGTAGGTGGGACTACAGGTGCGC -874

ACCACCACGCCCAGCTAATTTTTTGTATTTTAGTAGAGAGGGGGTTTCACCATGTTGGCC -814

ACGATGGTCTCCATCTCCTGACCTTGTCATCCGCCCACCTTGGCCTCCCAAAGTGCTGGG -754

ATTACAGGCATGAGCCACCGCACCCAGCCAAGAATCATTATTTTTAACTTGATGACTGAA -694

AATAATAATAATAATAGTTACCACTTATTTGCATGCTTCTATGTGCCAGGTAGTTGCTAA -634

CTATTTAAACTCAAATTCCATGAACTGTAGTGGAGGTTGTACTGGAATTTGATTCAGAAT -574

GACAGTGTCCATGATGGAGCAATAGAGGGCTCTCTATTTCAAACCATACCTCCTTGCTTT -514

TACCTCCTGCCTAAGTCATCAGGGGTTAGAAGGCTTTCCAGGTATTGGCCTCTTTCCTTC -454

ATTCCTAAACCAGATTGGTTGCTTATTTCCGTCAAGCTGAAACCAAAAGTAAGCAACCAA -394

AAAGCAACAAGCAAACAAAAGCCTTGTTACTCAATTAATTAAGAGTAGATTTTTATATTT -334

GATAGTAGGTTCCTTCTAAATATAGAAACTGAAAATAGAGCTATCTCCTTCAATTCTCCG -274

TTTTCTGTGTATTCATCCAGAATCCAGCCACCAACTGCCACAATAGGCAGCAATGGACTG -214

ATGTTCTTTAGGGAGGACGCGAATCTCGTTCCAAATGCTGGCCAGTCATTGGGTTTCTGC -154

AGCACTAGAAACATCTATGGTTGCAGGTCTGCAGTTTATCTGTTTTAAAATAGAAACAAA -94

GTTTCATTTCCCCCCCCCGCCCCGCCCCGTCAGCAGGAATTCAGCTAGCTTTAGTTTCAC -34

TTTCC CCTTTCGGTTTCCCTAGGTTTCCAACTTGCAAGGACA +9

>IFIT1B[Pan_troglodytes]

AACAATTCACAAGAGGAATTCAGAATAGCAGCTTTCCAAATTTTCCTTTTTCTCTGCTAA -918

TTGTGTGGGCAATTACATCAGCCTTGGGATCATATAAATTTTTCATTAAACATGTGAACA -858

TTGTTTGATTGACTCTCTGATGGTTGTTGCTTTGTAACAGATAATCTTCAAAATAGTAGA -798

AACAGAAGAAACATAAGGACAACTCAGAAGAACTATGTGAATTTGGATTTAGATTTTTAA -738

CCCCCATGACCCCATCACATTCTCTGACTACATTGCATATGTTTCTCTACTGACAAGCTC -678

AAACATTGTTGTTTGAACCACTAATGGTGAACAGTAAAGATCATCAAAACAGTCAATATG -618

CTTGAATAGGCTCCACTATAGCCAGGGTTGGCAGTAATGCAAATGCTGCTGGAGGATGTA -558

ACCAGTATGGGACCCTTCTTATAGACCAATATCTCAAAATTGAGGAGTTGTTTTGCCTAA -498

CACAGCCCCACAGAAGAAGACCTAACCATCTGCAACCTCATTCTTCCACAAATTGACAAT -438

GACTTTCTACAGTATTCTTTTAGGCACAAACCACTGACTCTGGTCTAAGGAGCTTCCATT -378

GTAGACGTCCTTGGGACCACCTGGCTGGATACATGTGATAGTAACAAGTTTGACAACAGA -318

GTCTGACATGATTCACAAAAATATACGTTCCTGTCTGCCACTGGTTTTTCCACAACACTG -258

AATGTCATCATAAAGTCCAGTTATCCTCTATCTCCTACACCACATTGACTCCAGCAAGGG -198

AATTTGCGTATCAAAACTGCTTCTCACACACCCATCCTCTTAGCTGCCAAAACTCAGCAT -138

GTATTTGTAATCAAAGCAGAGACAGCCCTATTTCTCTGTATCATTTTCACCCTGCCAATT -78

CACTAGGCATCAAGGATGTAAAAAACTGCCTGGATATACCTCCCATATATAAGCACCATG -18

CTTATCTGAGAAGCCCTAGAACTCTGTGGA +13

>IFIT2[Pan_troglodytes]

AATAATTATAATAGAAACTAGCATTTACTTATTACTGCCAGGCATTATTCTAAGGACATT -701

TCACGTACTCTCTATTTCATCCTCCCAACACCACAATAAACCAGTAGTGGTATTATTCCC -641

AATTTACAAAACCGAAGCAGAGACATTCAATAATTTTCCCCAGAGTTTCATAGCATAAAA -581

GTGGAGGCTGATTTCAAATCCAGATAGACAGACATCACTGCCCATGCTTCTAATCATGTA -521

TTCATTTATTCAACAACTACTTTTGGAATGCTTGCCATACCATGCAATGTGCAGGTTGTT -461

TTGCAGGGACTGGAGATTCAGCAATGATTCAGACTAAAATCCCCTGCCCTGTCGAGGCTA -401

AATAACTGCTCCTTACAGTGGCCTGTGGATCATATTGGTATAAGGCAGAGCAAAGATGGG -341

TTAATATGGTAATCACAAGGCAGGGCGCTTTGTAAATGTGTCCTCCTAGGAGTTGTGCTC -281

CCTTGATGCCATCAGTACATTCCACAAAGAGGAAGTTCTTCTAACTCACTACGCAGCTGG -221

GCTTGGCTCTTACTCAAGAGCATTTTGGGGTGAAAATATCCAAGCTTACCAGCCCTTGTG -161

CAAATCTGAGATGAAGAACTACATTAAAAGTAAAATTCACCTTAGCAGGAAGTGGGGTTT -101

GCTATTCTGTGTATATCTTTAACAGTATCTGCTGAAAAGAACCCTTTTGTTAAATAATTG -41

TATTACAAGTCCCCAACTTAATCTTTTCGAATATGAAATAAGAGAGGGACAGTGCACAAG +20

>IFIT3[Pan_troglodytes]

TCAATAGAAATTCAGTAGACAATCAATAGAAAGGAATGTCTGGGTTGTGGAGACCAAGGT -815

TTTATTATGCAGATGAAGCCTCCAAGTAGCAGGCTTCAGAGAGAATAGGTTGTAAATGTT -755

TCTTAATACACTTAAAAAGAGCATGTTCTGGCTGGGTGCAGTGGCTCATGCCTGTAATCC -695

CAGCACTTTGGGAGGCCAAGGTGGTTGGATCACCTGCTATCAGGAGTTCAAGACCAGCCT -635

GACCAACATGGCGAAACCTCGTCTCTACTAAAAATACAAATATTAGCCGGGCATGGTGGT -575

GGGTGCCTGTAATCCCAGCTACTCGGGAGGCTGAGGCAAGAGAATCACTTGAACCCAGGA -515

GGCAGAGGTGGCAGTGAGCCGCGATCATGCCACTGCATTGCAGCCTGGGCAACAGAGTAA -455

GACTCTGTCTCAAAAAAAAAAAAAAAAAAAAAAAAAGAGTGTGTTCTATTAGTAATTCCA -395

AAAGGAAAAAGGATGTAATGAGGCATATCCAGCTTCCCCTTCACATCAGTTTTCAGGTTA -335

ACTGTGGAATGCCCTTGGCCAAAAGCAGGGGTCCAGTCACATGGTTGGTGAGCATGGCCT -275

TAGAATTTAATTTTTTGGTTTACATTAGGAATGCTTCCACATTCTCTCAAACAAATTTTA -215

GTCACAGTTTTGAATAACGTACTCTGGCTCAGCCATTGCAGGTCTCAAGCCGTTAGGTTT -155

CATTTTCCTCCTCCCAACGATTTTAAATTAGTTTCACTTTCC AGTTTCCTTTTCCTTCCC -95

CTAAAAGCAATTACTCAAAAACGGAGAAAACATCAGCTGATGCGTGCCCTACTCTCCCAC -35

CCCTTTATATAGTTCCTTCAGTATTTACTTGAGGCAGACAGGAAGACTTCTGAAGAACAA +26

>IFIT5[Pan_troglodytes]

AAGAACCTACTGGAAGGAACCAATTCCGGACACACCATCACCTAGGTATTAAGCTCAGCA -879

TCACTTAGCTATACTTCTTGATGCTCTCCCTCCCACCACCCCCAACTCCAACAAGCCCCA -819

GTGTGTGTTGTTCCCCACTGTGTCCATGTGTTCTCATCATTCAGCTCCCACTTACAAGTG -759

GGAACATGCAGTGTTTGGTTTCTGTTCCTGCATTAGTTTGCTGAGGATAATCATTGGAAA -699

TACTTGCTCTGTATTTAGATTTCATAAAATTTGCAACTTAAAAAAAGCAGATTCGTATAC -639

CTAAGTTTTTCCAAATGTAAGTTTTCTATTATCAGTTTTTAAATTTATATTAAATTAAAA -579

ATCAGGTTCTTAATAGCCACATGTGACCAGCAGTTTCTGTATAAGACAGTACAACTGTTA -519

GAAGTGATTGCTTTAAATGAGACACTCGCTTCAGGCACTAAGTCTTGGGGGGTGGGGTGC -459

CAGAAGCTCAGTGATCAAGGTAGACAATATTTCAATGCAATATGTTTTTAAAAATTCAAA -399

ATCAGGCCAAGCATAGTGGCTCATGCCTGTAATCCCAGCATTTTGGGAGGCCACAATGGG -339

CGGAACAATATTTAAAGTTTAGAAAAATATTTGTGCCCAGGTGTTGGAGACCAGCCTGGG -279

CAACATGGCAAAACCCCGCCTCTACAAAAAATTAATTAGCCCGACAGGGTGGTGCATGCA -219

TATAGTCCCAGCCACTCAGGAGGCTGAGGTGGGAGGATCGCTTGAGCCCTGAAGGTTGAG -159

GCTGCAGTGAGCTGTCATCTCACCACTGCACTCCAACCTGAGCGACAGAGTGAGACCCTG -99

TCTCAAAAAAAAAAAAAAAAAAAAAATCAAAATCAATGCAAAAGATGAATTATGGACAGA -39

TATTGAGGTTTTGAATACAGACATTAACTCAATAAGGGTAAGGCCCGTGTCTGTTTTCTT +22

>IFIT1[Macaca_mulatta]

GCACCACCATGCCGGGCTAATTGTTTGTATTTTAGTAGAGACAGGGTTTCACCATGTTGG -793

CCAGGATGGTCTCCATCTCCTGACCTCGTCATCTGTTCACCTCGGCCTCCCAAAGTGCTG -733

GGATTACAGGCATGAGCCACCATGCCCAGCCAAGAATCATTTTTAACTTGATGACTGAAA -673

ATAATAAGAATAATAATCACCACTTACTTGCATGCTTCTATGTGCCAAGTAGTTGCTAAC -613

TATTTAAACTCAAATTCCATGAACTGTAGTTGAGGTTGTACTGGAATTTGATTCAGAAGA -553

CAGTGTCCATGCTGCAGGAATAGAGGGCTCTCTATTCCAAACCATAGCTCCTTGCTTTTA -493

CCTCCTGCCTAAGTCATCAGGGGTTAGAAGGATTTCTAGGTATTGTCCTCTTTCCTTCAT -433

TCCTAAACCAGATTGGTTACTTATTTCCATCAAGCTGAAACCAAAAGTAGCAACCAAAAG -373

CCTTATTACTCAATTAAGAGTAGATTTTTAGATTTGATAGTAGATTCCTTCTAAATATAG -313

AAACTGAAAATAGAGCTACCTCCTTCAATTCTCATTTTTCTGTGTAGTCATCCAGAATCC -253

AGCCACCAACTGCCACAACAGGCAACAACGGACTGATGTTCTTTAGGGAGGATGCGAATC -193

TGGATCCAAATGCTGGCCAGTGATTGGGTTTCTGCAGCACTAGAAACATCTATGGTTGCA -133

GGTCTGCAGTTCATCTGTTTTATCAAAATAGAAACAGTTTCATTTTCCCCCAAGCAGGAA -73

TTCAGCTAGCTTTACTTTCACTTTCT CGTTTCGGTTTCCGAGGTTTCCAACTCGCAAGGA -13

CACACCCACAGCTTACACCATTGGCTGCTGTTTAGCTCCCTTATATAACCCTGTCTTGGG +48

>IFIT2[Macaca_mulatta]

AGTGGGGGCTGATTTCAAATCCAGATAGACAGACATCACCACCCATGCTTCTAATCATGT -689

ATTCATTTATTCAACGACTACTTTTGGAATGCTTGTCACACCATGCAATGTGCAGGTAGT -629

TTTGCAGGGACTGGAGATTCAGCAATGATTCAGACTAAAATCCCCTGACCTGTCGAGGCT -569

AGATAACTGCTCCTTACAGTGGCCTGTGGATCATATTGGTATAAAGCAGAGCAAAGATGT -509

GTTAATATGGTAATCACAAGGCAGAGTACTTTGTAAATATGTCCTTCTCCGAGTTGTGCT -449

CCCGTGATGTCATCAACACATTCCACAAAGAGGAAGTTCTTCTAACTCACTACCCAGCGG -389

GGCTTGGCTCTTACTCAAGAGCATTTTGGGGTGAAAATATCTAAGTTTACCAGACCTTGT -329

GCAAATCTGAGATGAAGGACTACATTAAAAGTGAAATTCACCTTAGCAGGAAATGGGGTT -269

TGCTATTCTGTGTATATCTTTAACAGTATCTGCTGAAAAGAACCCTTTTGTTAAATAATT -209

GTATTACGAGTCCCCAACTTAGTCCTTTCGAATATGAAATAAGAGAGGGACCGTGCACAA -149

GAGCAATGCCCCCAGACCCATCTTTAAGTGAAGCGCCAGGCGGATAAAACATCCCTCTTC -89

TGCTGCCTTCTTTCTCTGATCGCAACTCAGCTCCCGAGGGAAAAGAGTGCTCATTTCCCT -29

GTAAAGAGTCCTGCCAATTTCACTTTCT AGTTTCACTTTCCCTTTTGCAACGTCAGCTGA +32

AGGGAAACAAGCAGAAAGGAACCAGAGGCCAGTTGTATATATAGGTTTCTTCAGCATTTA +92

TTGGTGGCAGAAGAGGAAGATTTCTGAAGAGTGCAGCTGCCTGAACCACAGCCCTGCCGA +152

ACAGCTGAGAATTGCACGGCAACCATGAG +181

>IFIT3[Macaca_mulatta]

GATTCAAAGATTTTCTGATTGATGATTGGTTGAAAGAGTTATTATCAATAGAAAGGAATG -703

TCTGGAGACATTCAGTAGAAATTCAATAGACAATCAATAGAAAGGAATGTCCGGGTTGTG -643

GAGACCAAGATTTTATTATGCAGATGAAGCCTGCAGGTAGCAGACTTCAGAGAGAATAGG -583

ATGTAAATGTTTCTTGTAACACTTGAAAAGAGCATGTTGTGGCTGGGTGCAGTGGCTCAC -523

GCCTGTAATCCCAGCACTTTTGGAGGCCGAGGTGGGTGGATCACCTGAGGTCAGGAGTTC -463

AAGACCAGCCTGACCAACATGGTGAAACACCATCTCTACTGAAAATACAAAAATTAGCTG -403

GGCGTGGTGGTGTGTGCCTCTAATCCCAGCTACTCGGGAGGCTGAGGCAAAAGAATCACT -343

TGAACCCAGAAGGCAGAGGTTGCAGTAAGCTGAGATCATGCCACTGCATTCCAGCCTGGG -283

CAACAAAGTAAGACCCTGTCTCAAAAAAAAAAAAAAAAAAAAAGAGTGTGTTCTATTAGT -223

AATTCTAAAAGGAAAAAGGGTGTAATGAGGTATATCCAGCTTCCCCTTCCCATCAATTTT -163

CAGGTTAACTTTGGAATGCCGTTGGCCAAAAGCAGGGGTCCAGTCACGTGGTTGGTGAGT -103

ATGGCCTTAGAATTTAATTTTTTGATTTACATTAGGAATACTTCCACATTCTCTCAAACA -43

AATTTTAGTCATGGTTTTGAACAATGTACTCTGGCTCACCCGTTGCAGGTCTCAAGCTGT +18

TAGGTTTCATTTTCCTCCTCCTGACGATTTTAAATTAGTTTCACTTTCCAGTTTCCTCTT +78

CCTTCCCCTAAGAGCAATTACTCAAAACATCAGCTGATGCGTGCCCTACTCTCCCACCCC +138

TTTATGTAGTTCCTTCAGTATTTACTTGAGGCAGACAGGAAGATTTCTGAAGAACAAATC +198

AGCCTGGTTACCAGCTTTTTGGAACAGCAGAGACGCAGAGAGCAGTCATGAG +250

>IFIT5[Macaca_mulatta]

AGATTTTGAATACAGACATTAACTCAGTAAGGGTAAGGCCCGAGTCTGTTTTCTTTATTG -708

TTATATTGACAGTACTTTGAACAAAGTCTGGCTCTTACAGTTGTTTGTCAACATCTGCCG -648

AATGAATTCAGAGGGGACAAAATGTTTAAGGTATGGGTGGGTGTTTGACATTTTCTGCCT -588

AGATCCCAATACCACTCTCGTCTTTATTGGGTACTCATTACACAAAGCACCATTGGCGAA -528

GGTTGGAAGAGAAGCACTCTCCTACGATTGTGGGTAGGAGTGGATTTTTAAAGCTATTTG -468

GTAATATCCGTCAAAACTTAAAATGTATATAGATTTTAACTCCAAATTTCTCTAGCTTAC -408

ACCCAACAGAAATAACAGCATAGATGTATAGCATTACCGTGTTTCTTGTTGAATCAAAAG -348

ACTGGAGTGGAAGCAGGGACTTCAGTTTCCGCCTGTAATCCCAGCACCTTGGGAGGCAGA -288

GGCGGGTGCATCGCTTGAGATCACGACTGAGTGACACAGTGAGACCCTGTCTCAAATAAA -228

CAAAGAGTTAAACGGGCAGCACGCGAACTCAGCGGACTCAGAGAGGCAGATAAGAGCCGA -168

GACCAAAGTGCAGGAGCCCAGCTGCAAATTTAACTCCGAAAAACCCTGGACTTCGCAAAC -108

GTCTGGCCGCGGCTCTTGAGCTCTTCTATTAAGTTTCGGATTCTGAAGCTTCGTTAAGTT -48

TCCATTTCTTAAGTTTCGTTTTCTGAGAGCTCGGGCATCACATTCAATTTCCAGTTTCCT +13

>IFIT1[Mus_musculus]

ACAGTGTGAGCCCCACTGTCTGTAGTTCCCTAGATTGCTATTAGCCCTTAAAATGGTATT -959

TATTTTACGTGTATGAACACACTGTAGTTGTCTTCAGACACACCAGAAGAGGGCATTTAA -899

TCCTATTACAGATACTTGTGAGCCACCATGTGGTTGCTGGGAACTGAACTCAAGACCTCT -839

AGAAGAGCAGTGAGTGCTCTTAACCACTGACCCATCTAGTAGCCTTGCTATTAGTGGGAT -779

AGCATCAAGGCTCTGAATAACCATCACCCTTTAGGGATTCCATGTTTGGTTCTTGTTAAC -719

ATGACTATGATTGGTGGAAAGAACCCTTTAGAGTCACCTCAGGAGAAGTTAACACAAAGC -659

ATGGCTCTACAGCTCTCTGGGCAATGGGAAACTGTCCTGGTTTATAAAGACTGTATTCCT -599

AAGGCTTCGGATAGGCTGGGAAGCTATGGACCAGTCAATGCAGGTGAATAATGACATCTG -539

TGGAAGATGCTAAGGATAGACACACAACAAACCAGCTAGTATCTTCTTCCACTTCCACAA -479

TGCATGAGAACTCACTCTTCCAGTCCCAAACTTTCCCTTTGCCATCCTAAGACCCCCTAG -419

TGCCACATGGGAAAGTCTCACTTCCAAGGAGGAACTGCAGACCTTCAGTTGCTTGGAAGA -359

AAGAACAACACTGCTTCTGAAGATGCCAGTCTGCTTTCCATTTTATCTGTATCACCTCCA -299

TCAGCCCCTACCTAACAGATAAGAGTCTCTGGGTGGCAGGGATGTCTCACTCTCTCTCTC -239

TGGACAGCAGTCTGGATCCGACATGTCTGTATCCGTTTCAGAGCCTTCTCCTCATCGATT -179

TCAGTGGAGAATGCAGTAGGGCAAACCCCACAGTGCCCCACAGTGCGTCTCCCTGGATAA -119

ACTGCAGGCTTCAGTTTCACTTTCCAGTCTCAGTTTCAGTTTCTCACTGCTGACTGAAAA -59

GAGCACACCCCCTAGCTCACCCAGCCCTTTAAAAAAAACCTGAGCTTCCAGTTGGTGTGA +2

CAGTCTGCAAGAGA

>IFIT1B[Mus_musculus]

ACCCACCATACCCTAAGCCTCAGTTGTGTGCCCACCGAGTCTCTGGGTCTCTACTTGCTC -992

TACCTTATCAAACTTCCTGACATGGCTTTATATAACATATATTCATATATATTTATTCAT -932

ATATATTTAGTTTCTTGGCCAAAAGGTTTTAATACCTCTTGTCAGAGAGAATAGAATTGA -872

CACATTTTTGTTTCTTAATCCTGGGGGGGGGGGGGGCGCGGGGAGGGAGGGAGGGAGAGA -812

GGGAGGGAGGGAGAGAGAGAGAGAGAGAGAGAGGTGTTGCCCTGGCTATTTGTTTAGCCA -752

GCCAGGGATGGGAATGAATGCGCCTGGAACAGAAAGCTTGAACAGCATTTCAAGAGGTCC -692

ATTGAGGGATAGGCTGGAAGATTCTGAGGACATGCTGGAACATTCTAAGGACTATGTCCT -632

CAGAAGATCAGAAGGAGCATACTCTAACCTGCTGCTCACTTTTGGAGCACCAAGTGGGCA -572

CTGGTGGTCCTTTATTCCATTCCAGAGGGCCTGTTTGGTAATCATCTTTGTTTGTGAGCC -512

GAGCATGCACTAAATTCTCTCCCAGGTACGTCCTACTACATGCTGTGAGACAGCCTTAAA -452

AATCAAAACAAGCTTCCAGTCACACATCCAGCACCAGGCACACAGCTTAGTGGGGGCATT -392

AAGCAAACTCCCTGGACCCTCAGGACAATTAGCATCCTTGTTCCCCATCAGCAGTAGAAA -332

GAGAAGCTAGAGGCACACTCAGAAACAGCCACTAGCAAACGTCTGTGTCCTGGGTTCCCT -272

CAGAGTGGAGAACAGGGAAATCCTTACCCCATGGTTGCTGTAAAGGTGGTAGCTGTTCTC -212

TGGGTTTTCAGCAGAGCAGAACTCCTCAGAAACCTGCCTTGACTCTCTGCTCTCTTGCAG -152

ACTGTCACACCAACTGGAAGCTCAGGTTTTTTTTAAAGGGCTGGGTGAGCTAGGGGGTGT -92

GCTCTTTTCAGTCAGCAGTGAGAAACTGAAACTGAGACTGGAAAGTGAAACTGAAGCCTG -32

CAGTTTATCCAGGGAGACGCACTGTGGGGCACTGTGGGGTTTGCCCTA +17

>IFIT1C[Mus_musculus]

CTAAGTGGATTAAGGAACTCCACATAAAACCAGAGACACTGAAACATATAGAGGAGAAAG -993

TAGGGAAAAGCCTCGAAGATATGGATGGGTACAGGGGGGAAATTCCTGAAGAGAACAGCA -933

ATGGCTTATGCTGTAAGATCGAGAATCGACAAATGGGACCTTTGTTGTTTTGAAACCAAC -873

ATACCAAATACAAAATGAAAAAAACCAATCACCTGCAAGCTCATTCTGCCATAAATTCTC -813

TCTCAAGCCTTCTGTTATACATAAACTTTGACCTTACTGCTATATGTGATCTTAAAAAAT -753

AATATGGCATGTTCTATGAATATATATATCCCTGCCTCTGGTCTTCTTGCTTCAACAATG -693

GGTATAAAACCTCAGGAATTCCCTGACACAAACACTACATGGAGCCCAGCACTAACATTG -633

CTTCCCACAACAATCTCATTAGGTTCCTAAGGCAAGCATGTCTTTGTTGCTGGGATTTCA -573

AACCATAAAGCCATATAAAAACTCTCAATACATTAATTATAATTATATCTATATGTCTAG -513

ATTTGGCAGATCAAACATTATTCTAGGTTATTCCCCAACTATAAGACATCTTTCCAATTG -453

TGTTTCTCCTGGACGTGTGGTTCTGGCTCATCATGGCTCATCCTCCTTTTCCTCTGTCCT -393

CTCTCCTTCCCCTCCTGCTCTCCTGCCATCTCTCTTCCTGCACCCCACTCTTGAACCTCC -333

AGTCCCTTCTTTCCCCTTCTCTGCCTACCCACAAGCTCCAGCCATTTTTGCCTAGTTAAA -273

ATGGGAAGAAGTTTCATATCACCAGAGTACATGATGGACAGACAGCTCCTGGGTGCTTAG -213

GGAGGGGAATCACTCTTAAGGAAACAGAATTAACAGCAACATACAAACAAGCACCAGACC -153

AACCTACCACACTTCCTCCTTATAAAAGAGCAAACAGCACTCTGTAGGTTTCATTTTCTG -93

TAGGTTGGAACATTGCCAGTTTCAGTTAGGAACTGAGGGTGTAGCAAACCTTCCCTATAT -33

AAATACCTTGCTTATCCTAGAATCCCTAGAACACTTTTGATTGAGTTTGGAAGCA +23

>IFIT2[Mus_musculus]

TGTAGTGTTAAGTACCACTATTAGATTAAAATGTCTCATCGCCTGTGAAGAGAAGGAGGA -1034

GGAGAGGGAGGGGGAGGGGGAGGGGGAGGGGGGGGGACTCAATTTAGAAGAGAAACTAAA -974

TTGACAAGACCGAAGTGTTTAACCACCAGGCAACAGGGAGGGGTATGATGGATCCTTAGA -914

ATGACAGTTCACTGCTAAAACTCTTCCTTTCCCCATCCCCTATCTCCTTTCAGTCCTAGG -854

GACAGAAGCCAGTGTCATGGGCAAGAGCTCTGTCACTGAGCTACATCCTAGCCTTGGAAC -794

CTCACTTTCTTTTAAAGGACTTTGAATGTCATTATCTTTCATTCTATCCATCTATCTGGA -734

TCTATATCTATCTATCTATCTATCTATCTATCATCCATCCATCCATTTATCAAGGGTGCC -674

ACACGGAGGTCAGAGGACCACTTGCAGGAGTCGATTTTTACTTTCCCTGGGATTAAAATC -614

CCATCCCAGCTCAGATCCTAGGGCTTAAAGTCAAGTGATATAAGCCCCTGAAGTCATCTC -554

CCCTAAGCCAGTTGAGTCTCAATTTCAATTTAAATCCAGGAATTCAATTCAGGATCATGT -494

CCCTCACTAAGATGGACTATTAGGAAACTGGAAATGAGTGAACCATCCTCTAAGAATCAA -434

CTAGGTATTATCTCTGCTGTCCAGAATATTACAGTCTGCGAATTAATCCGCTTCAGTTAG -374

CAACATCAGTAAACATTAAAAATAGGAAAGGACAGAAAAGGATCCTGTCTGGTCTTGCAT -314

TGTGCAAGGAGAATTCTATGCTAGAGCTGGGCTGGAGCACCAAGACCCAGAGGCTGGAAA -254

ATTCATGGGAGCTTCCTGGAAGAATGGATAAAACCGTCTCTCTCCCAATTCCGGAAGTAT -194

TTCTGCTGCAAACTCTCAAATTTAGCATCTAGGGATAATAAAGCTAAAAGCGGCGGAGAA -134

GGGCACCCTCTGGCGACAATAGCAGGACACAGTGGGAGGAAGCAGCGCTAAGATGGAGTA -74

CCACCCCAAAACAAAGCTCCCTGGGAGCTGTAGTCGAGCCAGGTTGCCTGGGTGAGTGCA -14

GTTTCCCGCCCTTAGTGATGTGGGGACCCAGCGAGAT +24

>IFIT3[Mus_musculus]

AGGAAATGAGACCAATGAAAATTGTATCCTTTCTGTGGCTTTGCTACCTGAGGCTTCCAA -879

AGTTTAGGCTTAAGGCAAACACAGTGGGTCTAGCAGGTATGTGCTTACCTCTTATGCCAT -819

TAACAATCTCAAGTTGCTAAGTGAAAGAAGGACCCAGAATTCCACAGCTGACTAGGTTCA -759

TGCCATGCTTAGCAAATGAGCAGTGAAAGCCACGCTTTAAGGAAATCATGAAGCTAAGCA -699

CCCAATCCATACCTAAGGATTGGTCCTCCATACCTCCTTCTACTCATGCATGACTTCATA -639

ACTCAAAGCAGAAGTCTTTCACTTAGTCACATATTTGTTCTCCCACCCAGGAAGCAGTGA -579

TGCACATGTTACCAATTCTATTCAGGGACTGGGTGTAAAGTAAATGATCACCACAGACCT -519

CTAGGTGTGAAACAGCACAGCGAATCCCCCTGGTGTTTTGCTACTTGAATTCAATGTAGA -459

ATAAAATGAAAACATTGGTTTGATTAGACTCTGGCAAAACTCCAGAGCTAGTCACATGGA -399

CGTAGTCATTAAAAGCTGGATTGAAACCTGTGTACCACAAGGGAACTCAGAGAGGGAACT -339

CACTATCACTTCTTTCAAAATATTTTAGAATAGTTTCTTTCGTGTGGCCCCAGAAACCTC -279

TGCAGAGTGCACTAGAGACTGTGGAAAACACTCTTTTCCTCCCAGAGGGACTGTCAGGCT -219

GGAGGAAATGCCTCGCCACCCTCTGACACCAGAGGCAGAGTCCAGAGCTTATCCAATGCT -159

GTGGCCAGGACATAGAAGGTCTCAGTGGTAAGTTTCACTTTCCTCTTCTGGTTGATTTTA -99

AGTCTGTTTCAGTTTCTACTTTCCTCTTCCCTTTCACAAACACTGAGTCCTTAGAAGCAG -39

CAGAGCACCAGCTGATTCACCCCCCCCCCCACTGCCCCCTTATATAATCCTCTGAGTCTC +22

>IFIT3B[Mus_musculus]

TACATTAGCATTCCAAGTCCCACTAGACCAAAATCAGGCATTGTGCTCTGAACCACTAAA -997

GAAAATGAGATCAATGAAAGTTGTATCCTTTCTGTGGCTTTGCTACCTGAGGCTTCCAAA -937

GTTTAGGCTTAAGGCAAACACAGTGGGTCTAGCAGGTATGTGCTTACCTCTTATGCCATT -877

AACAATCTCAAGTTGCTAAGTGAAAGAAGGACCCAGAATTCCACAGCTGACTAGGTTATC -817

ATGCTTAGCAAATGAGCAGTGAAAGCCACGCTTTAAAGAAATCATGAAGCTAAGCACCCA -757

ATCCATACCTAAGGATTGGTCCTCCATACCTCCTTCTACTCATGCATGACTTCATAACTC -697

AAAGCAGAAGTCTTTCACTTAGTCACATATTTGTTCTCCCACCCAGGAAGCAGTGATGCA -637

CATGTTACCAATTCTATTCAGGGACTGGGTGTAAAGTAAATGATCACCACAGACCTCTAG -577

GTGTGAAACAGCACAGCGAATCCCCCTGGTGTTTTGCTACTTGAATTCAGTGTAGCATAA -517

AATGAAAACATTGGTTTGATTAGACTCTGGCAAAACTCCAGAGCTAGTCACATGGACATA -457

GTCATTAAAGCTGGATTGAAACCTGTGTACCACAAGGGAACTCAGAGAAGGAAACACACT -397

ATCACTTCTTTCAAAATATTTTAGAATAGTTTCTTTCGTGTGGCCCCAGAAACCTCTGCA -337

GAGTGCACTAGAGACTGTGGAAAACACTCTTTTCCTCCCAGAGGGACTGTCAGGCTGGAG -277

GAAATGCCTCTCGACCCTCTGACACCAGAGGCAGAGTCCAGAGCTTATCCAATGCTGTGG -217

CCAGGACATAGAAGGTCTCAGTGGTAAGTTTCACTTTCCTCTTCTGGTTGATTTTAAGTC -157

TGTTTCAGTTTCTACTTTCCTCTTCCCTTTCACAAACACTGAGTCCTTAGAAGCAGCAGA -97

GCACCAGCTGATTCATCCCCCCCCCCCCCCCGACTGCCCTCTTATATAATCCTCTGAGTC -37

TCCACTTAGAGTGGACTGAGATTTCTCAGCCCACACCCAGCTTTTCCCAGCAGCACAGAA +24

ACAGATCACCATCATGAG

>IFIT1L[Rattus_norvegicus]

TCTCATATGTTGATGAAAATATACCTCCATGTCTCTACTCTTTATGTTCCAACACTAGAT -1107

GTAAAGCCCTAGTTATATACTCACACAAAGGCTGCATGGAGCCCAGCATGAACATGGCTT -1047

CTCACAAAAATCTCATCAGATTCCTAAATCAAGCATATCATTGTTGCTGGAATTTCCAAC -987

CCTAGTAAGCCATATAAAGAACACACAATCCAGTTATTATAAACTAACCCCTATATTCGA -927

AAGTGATTGTAGGTTGCCAGTGCCCTGGTCATTGGTACTGAAGGGAGAGGATGCAGAAAG -867

TCTTTCCCCATGTTTGGAGGACCCAACCTTATCCTTGCCAAACCTTGGTGCTTTCCCACA -807

TTGCCCACTGCCTGTCCTTCTAAAAAAATAATAATTAAAGCTATATGCCTAGATTGGGCA -747

GATCTAGCACTCTACTAACTTGTTCCACAGCTATGAGACTCCTTGTTATTTGTGGTTTCT -687

CTTGGCCAGGTTGTTCTGGTCCAGTGTGGCTTCCCCTTTTTCACTGTCCTCTCTTCCCCT -627

CCTGTTCTCCCTCCCCTCTCCCACCTATCCCCTACTCTTAAACCTCCAGTCCTTTCTTTT -567

TCCTCTTATGTCTAATCACCAGCTCTAGCCATTATGGGCCACTTAAAATTCAATGTTCCA -507

AGGGGAAGCTTACTGACTGCCCATGGGAGGACAGAAGTCTGATCCAGAGCAGAGACTGGC -447

AAGTAGACAGAAAACAATAATGATTTCAGAGCAGTGGGAAAAGGAGAGGAGCCAGGTTTT -387

GATTTGATAAGTTAATAGAAACTTGTTGGGTAGAAAGAAGAAACTAATATTAAAGGTGAT -327

GTTTTTGTTGTTTTCATTGATAAGTCAGAAGTTTCTTTAAAATACATATGACATCACCTG -267

AACTGTGGGGCAACCCTTCTTGAAGAAGCAGAATTAACACCAGAATACTAACAGTACCAG -207

GCCAACCCACCACTCTTCTTTACTATCAAAGCAGAAACAGCCCTCTCTAGGTTTCGTTTT -147

CTCTAAGCTGGAAGCTTGCCAGTTTCACTCAGGAATTGAGGGCGTGGCAAACTGATAATC -87

CTTCCATATATAAATGCCTTGCTCATCCTAGAACCCCTAGAATGCCTTTGAGTTTGGAAG -27

AGTCTCTTGGCCTGAATTAAAGTGCTATGGAACAAGTTCTTTCTCCCAGCACCATGAG +32

>IFIT2[Rattus_norvegicus]

CGGTCTGCAGTGACAGATGCTACACATCTGGATACACACATGTTAACTAGCGAGCAGCCC -769

CACCGGTGCATCCCAAGAGGGATGCCAGGGATATCTGGAATGCCTTCCAGGTTAAAGGAC -709

TATTTGCCTCAGAAATGTTAGTGATTATGACAGGTCAAAGGGGATCGGAGAATTTCGGGC -649

TCTGGAAAAAAGCAAACGTCCAAAACTTTTGGCTACTAATTATACTAGTTACTCGGTGCC -589

AGTCATAATTCTAGAGCATGCCCTCCATGGACTCAGTATTACTCTCAGTTCAAAGCAGGA -529

GACCCTGAAAGCGGGGGCTGAGAGTAATCTCTCCTGGGGAGCAGCATAGAAATGCAGATA -469

GATAAACATCAGCTTGGAGCTGAGTCCATTCTTTGAAGGTCTGCATTCGGAATCCCTGCC -409

CAGTCATGCAGTGTGCCCTTCTCCTTGAAGGAAAGGGGCTTCAGTTGAGCAGATAAAACA -349

GACACAAAGCCCTGCTCTGTGAAATAGAAACTCTTTCTTCCCTGGATCATGAGGATGCTA -289

GAGTGAGGATGGATATGTTGGTCTCTATGAGACAGGGTGCCGTGCACGCGCTTGCTTGCT -229

TGTGTGTGTGTGTGTGTGTGTGTGTGTGTGTGTGTGTGTTGCACACGCTTACACGCTTGG -169

CTCTTTTTTCAACCCTTTTGTCCATAAAAAGTGACATCAATATGACTTTCTGATTTCAAT -109

TTCTTTTCTTTGGGGTGGGGGGAGGAGTCAGGCTCTTTTTCAACCCTTTTGTCCATAAAA -49

AGTGACATCAGTGCAACTTTCCGGTTTCAGTTTCTCGTCGTCTTTGGAGTCAGCTGCACA +12

GAAACAGGAAA

>IFIT3[Rattus_norvegicus]

AAGCCAGGCTAACCAGACTCGGAGTGCTTATGGGCATTAATGTCATGACTAGGGGCTTTT -980

ATCAAACCTTTTACCAATCGTTATGGTGACAGTATGTGGAAACCTTTCTGGAACAGGTGC -920

TTCGATGCCAATGAGACCACATGAAAGAGAAGATATGCTGACTAATGGACCGTGGCTAGG -860

AGAGCTCCTGAAAGACCTCAGTCTTGAGTACAGCTTCATTTTCATACCATTCTCTAAAAC -800

AACAAGGTGGGGCAAAGAAGCAGGCAAGATTTTACAGAACCATGTGAGGCAACATGCAGG -740

TTGTGAAGAGCCATGAGCCAATGTTTTTAGTTGTTTCTCCAGGTCATTTTGTCTCAAGGA -680

GCAAGTGACTTCATGTTTAGCAGACGAACAGGAAAAAAAGCCTTCCTTTAAGCAAATCGT -620

TAAGTAAATCAGCAAATCAGTAGCTAAAAATTGGTCTACTCTACCTTCTACTTCCACATG -560

ACCTCATAATTCAAAGCAGAATTCTTTGACTTTATCACAAATCTGTTCTCCCACCCAGGA -500

GGCAGTTATGCACATAATAATTCCATTCCAGAACTGGTATACAGGAAATGGTCATCGCAG -440

ACCTCTAGGAACTAAAAGGCACAGCAAATTCCCCTGGTGTTTTGCTACTTGAATTCAGTG -380

TAGCATAAAACAAAAGCATTGGTTCGATTATCCTCCAGGAAAAAAACAGGATTGAAAACT -320

CTCTCCCACAAGGGAACACAGAGGGAAACTACTATTGTTCCCTCAAGAGATTTTAGAATA -260

GTTTCTTTCATGTGGCCCCAGAAACGACTGTCAGAGTACCCTAGAGACTTTGTGGAAAAC -200

CTTCTTCTTCTTCCCAGATGCAGTGTCACGCTCCAGGAAATGCCTCCCCACCCTCTGACG -140

CCACGGGCTCAGAGCTTACATAAAGTTTTGTGTCCAGGACATAGAAGATCTCAGTAGTAA -80

GTTTTGTTTTCCTCTTCTGGTTGATTTTAAGTCTGTTTCAGTTTCTAGTTTCCACTTCCC -20

TTTCACAAACGTGAGTCATGAGAAACAGCAGGAC +15

>IFIT1[Bos_taurus]

GACTCTGTAAGATTACACAGGCCAGGCTGAGGCAGGCCAAAGTGGATGCCATGGGCACCA -878

AGAGGATAACAGTCTTACACAGATATGATAATCATGTCTAGTGAATACGAACACTTTAGA -818

TGGCTACAACAGAGGGCATTACAAATAAGGAAATATTTTCATTCAGGCCCAACATGTTTT -758

ATTTGAAGTCTCAAGATTCTAGCAGATTTTCTTGGTTCCCATGAACAAATAAGCAGTTTT -698

AGGACTCTTTTTTTTAAATCACTGATCATTCTCCAGAAAGAAGGGTATGCAAAAGCACAA -638

AATAATTAGTAAGACTGATCTCTCAGAGAAGAGGAACAAATTTGAGTTAAGGCTCCAGTT -578

TACTGACTTGCAGAATTTTATACCATATAAAGAACAATGTCTGTTTCCAGATGAGCAAAC -518

AAAGGCACAGATTGAGGGCATGACTTTGGCCAGTTCACAGAGCCAATTTTCACAGGACTC -458

CCTGGCTCTAGGTTCAGTGGACTCTACATTACTGGCATCTCTTAGTTCCTGGGTTTCTAA -398

AAGGCCTTACAAAGTCTGAGAGGAATTTGTTGTCATTCAGTCGCTCAGTCATGTCTGACT -338

CTTTGGGACCCCATCAACTATAGCCCCCCAGGCTTCCCTGTCCTTCACCATCTGCAGGAG -278

CTTGCTAATTCCCGAAGAGAGGAATCAGTACATCAAAAAGCTGCTTTCCACCAGCCTTTT -218

CTCAATCCATTTTCAAAACAACAAAGGAAGCATTAGCTGTTTAAGTTTCATTTTCTGCAA -158

TTCTGCCTACTTTAATTTCACTTTCT ACTTTCAGTTTCCAACTTATCGTTGCAAAAACAC -98

ACCCACAAGTTACATCATTAGCTGTGGTTCATCTCCCATATATAGCCCTGACCTGGGGCA -38

TAAATTTATCTGAAAATCCGTAAGACGGACAGAACAGACCTCAGAGAAGACCATCCAGCT +23

AGACAAAGCATTGCAGAACTGCTGCCTAATTCACAGCAACCATGAG

>IFIT2[Bos_taurus]

GCCATGTGCCAGGAGGCATGACTTCAGGAACTGGAGATTCAGTACTGAGTAAAATAGAAG -916

AAACCCCCCCTGCCCTGTGGAAGTTACATAACTGTTCATCACAGGGGCCAGCAGGACTTC -856

CCAAGTGGCGCTAGCGGTAAAGAACCCTCCTGCCAATGCAGGAGACATAAGAGACGTGGG -796

TTTGGTCCCTGGGTCGGGAAGGTCCCCTGGAGGAGGGCATGGCAACCTACTCCAGTATTC -736

TTGCCTGAAGAATCCCCATAGACAGAGGAGCCTGGCGGGCTACAGTCCATAGGGTTGGAA -676

AGAGTTGGCTGAAATGACTTAGCACACACTCAGCATGCACAGGGGCCAGCAGGTCATCTT -616

GCTGTTAATAGTGGACAGAGTGAAGATGGGTTAACATTCCAATAATGAGGCAGCTGCTAA -556

GTCACTTCAGTCGTGTCCGACTCTGTACAACCCCATAGAAGGCAGCCCACCAGGCTCCCC -496

CGTCCCTGGGATTCTCCAGGCAAGAACACTGGAGTGGGTTGCCATTTCCTTCTCCAATGA -436

TGAAAGTGAAAAGTGAAAGCGAAGCCACTCAGTCGTGTCCAACTCGCAGCAACCCCAGGG -376

AGTGCAGCACCAGGCTCCTCCGTCCATGGGATTTTCCAGGCAAGAGTACTGGAGTGGGTT -316

GCCATTGCCTTCTCCAATAATGAGGCAGAGTACTTGGTAAAAGTATCCTTCCTGGAGCTG -256

GTAGCTTCCGTTGATGGCAGCTGATATTACCACATCAAAACAATTCACAAAGAACTTCTG -196

ACTAACTGTAGGGGGAGGGGGAGGAAGTCACCCTATCAGTTTGGCTCTCACTTAGCACCC -136

AAGATTCATTTCCTCTTAGAGTCTTGTCAATTTCAGTTTCC AGTTTCATTTTCTCTTTCC -76

TAAAGTCTATTTTGGCAAAGGAATCTGGTAATGCATTCATATATAAGTCTCTTAACATTC -16

CACTGAAGCAGAGGAGGAAGATTTCTGAAGAGCGCAGAAGCCCTAACTGCAGTCTTTCAG +45

AACAGCTGAGAAGTGCACGGCAATCATGAG

>IFIT3[Bos_taurus]

AGGGCACGGTCTCCTATCATTTGGTTGTTAAGAAAAAAAAAGGTAATTTAGAAGGTATCT -888

TCTAAAATGGCCTGGAATAACTTATAATTTTGCTGATTCGGTGTTTGCTAATAGATTAAA -828

TATGTATTTCATAATTTGATCATAAATCTACCTTACCAGTTCATGCATTTTCATTTCTGT -768

GGCTCTTTCTGATCTACAGAGTCAGGTGCTGAGGACGTCCTCCCAGCAGAGCCAGCATCT -708

GGTAGCTGGGCAGGGATGGGAGCTGAGGAAATGAGGCTAAGTGATGTGATTCAAGAACTT -648

TCCCAGAGGCTGGTCAAAGAGAAGACTGAAATCCAGGATTTCTGGGGTTATTTTCCTCTC -588

TTCCAGATTGTCTGCCTCACTGATACATTACATGATCTCTCATAAGTAAATGGAAAACTT -528

TTCAAAATACCTCCAGGGACTTGCCTGGTGGTCCAGTGGGTAAGACTCCACACTTCCACT -468

GCAGGGAGCATGGGTGTGATCTCCTGGTCAAGGAACTAAGATCCCACTGGCTACACAGCC -408

AAAAACACCAAACCAAAACAAAATAAAGGAGCTCCAGGACTATGCTCCTCAAAATGTGAT -348

ACAGGGGCTCCTACAACAGAATTTCCAGGATGCCTGTTAAAATGCAGACTTGCTGAGCCC -288

AGGTCCACCTCCAGGCTGCATCTGAGTCTCTGGGAAGAGAGCCTGTAATATGCACAGCAC -228

CCACAATGCCTTCTTTCAAACTCACAAGCAGTTACAGGAGCTGTGTAGAAGGGTACTCCC -168

AGCGGGGAACTGAGTTCAGCTCTGCACGTTCAGTTTCAGTTTCTCCTCAGCAGGAATCTC -108

ACTAGCACTGCCTCAGTTTTTTCCTTTTCTGTTTTTTAAAAGCACAGACCTAGCAGCTAG -48

CATCCTGAGTGGGAACCTGCTCTGCATTTGCTTGTGATCCACAAGCAGAAAATAAAACCT +13

GCCTGAACCCCAGCTTTTCAGAATGGCAGGCAAACAGCCATCATGAG

>IFIT5[Bos_taurus]

AAGCTTTTATGTGCCAAGTAGTTGCTAAGTACTGGATTGGCCAAAAATTCATTCGGGTTT -988

TTCCATCATATCAGCACTTACTCTTCTCAACCTCTTACCACAAGCACCATGACTCTGTAA -928

GATTACACAGGCCAGGCTGAGGCAGGCCAAAGTGGATGCCATGGGCACCAAGAGGATAAC -868

AGTCTTACACAGATATGATAATCATGTCTAGTGAATACGAACACTTTAGATGGCTACAAC -808

AGAGGGCATTACAAATAAGGAAATATTTTCATTCAGGCCCAACATGTTTTATTTGAAGTC -748

TCAAGATTCTAGCAGATTTTCTTGGTTCCCATGAACAAATAAGCAGTTTTAGGACTCTTT -688

TTTTTAAATCACTGATCATTCTCCAGAAAGAAGGGTATGCAAAAGCACAAAATAATTAGT -628

AAGACTGATCTCTCAGAGAAGAGGAACAAATTTGAGTTAAGGCTCCAGTTTACTGACTTG -568

CAGAATTTTATACCATATAAAGAACAATGTCTGTTTCCAGATGAGCAAACAAAGGCACAG -508

ATTGAGGGCATGACTTTGGCCAGTTCACAGAGCCAATTTTCACAGGACTCCCTGGCTCTA -448

GGTTCAGTGGACTCTACATTACTGGCATCTCTTAGTTCCTGGGTTTCTAAAAGGCCTTAC -388

AAAGTCTGAGAGGAATTTGTTGTCATTCAGTCGCTCAGTCATGTCTGACTCTTTGGGACC -328

CCATCAACTATAGCCCCCCAGGCTTCCCTGTCCTTCACCATCTGCAGGAGCTTGCTAATT -268

CCCGAAGAGAGGAATCAGTACATCAAAAAGCTGCTTTCCACCAGCCTTTTCTCAATCCAT -208

TTTCAAAACAACAAAGGAAGCATTAGCTGTTTAAGTTTCATTTTCTGCAATTCTGCCTAC -148

TTTAATTTCACTTTCT ACTTTCAGTTTCCAACTTATCGTTGCAAAAACACACCCACAAGT -88

TACATCATTAGCTGTGGTTCATCTCCCATATATAGCCCTGACCTGGGGCATAAATTTATC -28

TGAAAATCCGTAAGACGGACAGAACAGACCTCAGAGAAGACCATCCAGCTAGACAAAGCA +33

TTGCAGAACTGCTGCCTAATTCACAGCAACCATGAG

>IFIT1[Sus_scrofa]

ACACCAACAACAGCTTGGCAGATCAGTAACAGGAGCCTAATCTATACCCAGGACCTTAGT -925

CTCATCTCTTATTCTTCTCAACCTCTTACCACAAGTACCATGACTACACAATACAGACCA -865

GGTTGAGGTAGGCCAAACCTGGATGCCATGGGCACCAAAAGGACAACACTTCTGCACAGG -805

TATAATAATCACATCTAGTGAATAAGAACACTTTAGATGACTATAATAGAGACTGTTAAA -745

ATATTTAAGTTTAGAGAAAATATTTTGTAAATAAAATATTAAAATATTTTAAAAAGGAAA -685

TATTTTCATTAAGTCCTATCATGTTTTGTTTGAAGTCTTAGAATGCTAGCACTTACATTT -625

TCTTTGTTCCTATGAATAAAGAAGAGATTATAGGATTTTTACTATCACAGACCATTCTCC -565

AGAAAGAAGGGTATGCAAAGGCACAAAATAATTAGCAAGACACAACCTCCCAGAAACAAA -505

TTTGGGTTAAGACACCAGTTTAGTGATTTGCAGAATTTTAATACCATAAAAAGAACAATT -445

TTTCTTTCCAGATGAGCAAACAAAGGCACAGATTGGGGGCATGACCTTGGCAAGTTCACA -385

TAGCCAAGTTTTACAGGTGTCCCTGGCTGCAGGTTCCATAGACTCCACATTACTGGCACC -325

TCTTGGCTCTCAGGTTCCTACAAGGCCTTAATGACCTTGAGAGCAGAATTAGCATATCAA -265

TTAAATTGCTTTCCACCAGCCTCTTCTAAATCAGCTTTCAAAACAGCAAAGGAAGCAATG -205

CCTGTTAAGTTTCGTTTTTCACAACACTGCCTACTTTAATTTCACTTTCTACTTTCAGTT -145

TTCATCTTGGTTTTCAATTTATCTTTGCAACAACACACCCACAGATTACATCACTAGCTC -85

TTGTTTACCCCACTTATACGTACAGCCCTGCTCTAGGGCTTAAATTTATCTGGAAATCCA -25

CAAGACAGACTAGACAGATCACAGAGGAGCCCATCCAGCTAAACCAAACACCGCAGAACT +36

GCTGCCTGACTCACAGCAACCATGAG

>IFIT2[Sus_scrofa]

AGGATATAAATAAGGATAAGGCAAATTTCCTCTATCAAACAAACCACTATGGCTGCAGAT -905

GGTGGTGTGCAGATGCTAAATCCCAAGGTGACCCACGGGAATTTAAGAAGTTGAGCTCAG -845

GGAAGCCAAGAATGTGTAGAGATGTTAGGAATGTTTGGCTTAGGTTAGCGATCATGAGAG -785

ATTGTGGAATGTCAACCAAAGTTCTGAAAGAGGCAAATATCACAAGCAAACCTTCTGGCC -725

CCCTTGTGTTAATGACTTTAGTCACTAATTATTATAAAAACTAACTTTTTAACTATGTTC -665

TGGGCATTATTCTAATGGCATTTCATGTATTAACCCCTTTGATCCTCACAACACCACATA -605

CATAGTGGTAGGATTCCCATTTCACAGAATAAACTAAAGCAGGGAGACCTTCAGTAATTT -545

CTAGTGTCACAGCATATAAACGGAGCCTGGTTTCAAATCCAGATTGATTGGCATCAGAGT -485

CCATGGTTCTAATCATGTATGTATTCATTCAACGACTTTTTGAATGCCTATCATGTGCCA -425

AGTATTATTATTGCGGGGACTGGAAATTCAGCAGTAAGTGAAACAGATGAAAACCCCCTG -365

CTGTGTAGAAGTTATATAACTGCTCCTCACAGGTGCCTGCAGGTCACCTTGGTGGTAAGG -305

GTGGACAGAGTAAAGATGGGTTAACATTGCAATAATGAGGCAGAGTGCTTAGTAAAAGTA -245

TCCTTCTTCCAGTTGGGCTCCCTTGATGGCAGAGGAAATTCACAAAGAAATTCTAACTCA -185

CTGCAGGGGGTGGGGGAGGAGGGTAACCCATGGGCTTGGCTCTTATTCAGCTCTTACTAA -125

GATTCATTTCCTCTTAGAGTACTGCCAATTCACTTTCCAGTTTCAATTTCTCTTTCCTAA -65

AGCCTGTTTAGGCAAAGAAATCAGGTGCTGCTTCCATATATAGGTCTCTTTAATATTTAC -5

TTGGGACAGAGGAGGATTTCTGAAGAGCACAGTAGCTGGAACTGCAGCCTTTCAGAACAG +56

CTGAGATGTGCACA

>IFIT5[Sus_scrofa]

TGTCTTTTTGATGATGAATACTTTTTCTCAAGGTTTGAGAGTTGTCTTCGCCCTCTGCAT -789

AACCAGAAACAAGATGCAGAAGACAGAAGGCAGGACTTGTCGCTTGTCTGTTTTATTCAT -729

TGCTGTATTGACAGGACCTTGAACAATGTCTGGCTCTTAGCAGTTGTTTAATAAAACTTG -669

AATGAATGACTGAATTTGAAAGGGACAAAAATTTTAGTGTATGGGCATTTGATACTTTCT -609

ACTTAGATTCCTAATACCACCCCATCTTTACTGGGACCTTATTATAGAAACACAGTTGGC -549

AAGGACATGGAAGGCAAAGGAACTTTCACACAATTTTGGGTGGGGGTGCAGAAATGTCTT -489

CACAAAGCTTTTCAGCAATGGCTACTAAAACTCTGAATGCTTACACATTTTGATCCCACA -429

ATTTCTCTAGATTTACCCAATAGAAATTATGGCTCAAGTGCACAGCATTGCACCTATGTT -369

TGCTGAATCAAGATTTCAGTTCAGCTTAGGATGCACAAAGCTGTCAAGAGCATCGCCGTT -309

CCCCAACAAAAATAACGCTTAACAATCTCCATTTTAGGTAAAACACCTCCCCCCCCAAAA -249

AAAAAAACATAAAACAAAACAAAAAAAAACGTTATCGAGAGACACGCGAATTTAATGAGG -189

CAGGTAGGAGCGAAGAGAGAAGTCCTAAAGCCCAGCTGCAAATTTCACCCTGGATGAACT -129

CCACCCCGTCGCGCGCTGCGCAAACCTCTTGCCTCGGTTCCTCAGCCCGGTCGGGGGCCC -69

CCCCAACTCAGCGCTTCCAGAACTTTCTGTTTCCCAAGCTGGACTCCGCCTGTCCGGGGA -9

TCAGATTCACTTTCTAGTTTCCTTTTCCCGCTCGGTCCGCGGTTGACAATGACTGCAGCC +52

>IFIT2[Equus_caballus]

AATGCCCCAGCCCTAGAAGTAAGTGCCATTCCCAGTAGTGGTGGCAGTAGTAGGTGTAAT -968

GGCTAGTCTTTGTTGGGCATTTACTATGTAACAGGCACTGTTCTCAGGGCTTTATATATA -908

ATGGTCCATTTAATCTTCATAAAATCCTGTGTGGTAAGAATTAATATAACTCCCATTTTA -848

CCAGTGAAGAAATTGAGGCCAAGAAAGGTTTCTAGCTCTATCACTTAGCAGCTGTTAGAC -788

TTTGACTCCTTTCTTCCCCCTAGATAGAATTAGTCTTACCCTCTCTCCTTGCTCTTTTCT -728

GCTGTAGCATTTATCACACACTATCTAATTTAATTATTTATTATTGTATTAGTTGACTTG -668

GGCTGCCAAAACAAAACACCACAGACTGGGTGGCTTAAACAATGTAAATTTATTTTCTCA -608

CAGTTCTGGAGGCTGGAAGTTCAAGATTAAGGTGCCAGCAAAGTTGGTTTCTGGTGAGGG -548

ATCTCTTGCTGAGTTGCAGAGGGACACCTTCCTGCTGTATCCACACATGGCCTTTCCTCT -488

GAATGTGCTTGGAAAGAGAAAGAGATAGGACACCAGTCCTATTGGATGAGGGCCCAACCT -428

ATATATCTCATTTAACCTTGATTACCTCCTTAAGGTCCCTATCTCCAAATACAGTCACAT -368

TGAGGGTGAGGGGTTCAACGTAAGAATGGGTGGAGGGGCACAAATCAGTCTATAATACTT -308

TTATTGTCTTCTCTTCCTGCTACAATATAGCTCCATGAATACAAGGATCTTTGTGTACTG -248

ATGTATCCCTAGTGCCCAGAATGGTGCCAAGTATATTCTAGGTATTCAATAAGTACTGGG -188

TGCTGCTGTAGGGAAAAAGTCCTCCAAATACAGAAGGTAGGAGAGAATGAGGTGTACGGG -128

AAAAGAGAGAAGGTACAGGAACTAATAGGACGAGCTGTGGATCATTTAAAGAAAGCTGAT -68

GAGATCAATGGAAATCTCTCTCATGCCTGCTCCTATCTTGCCTGCCTCTATGCACAAGCA -8

GGGCAGTATG +3

>IFIT3[Equus_caballus]

AAACTAAATGACTGCCTTAGACCTCCAAGATTTAAACAGCTTGGAGTTCACTCCAAGGTG -855

GCTTCCTAGATGAGTATGGAGCAAAAAGGAAATTCAGTCTCAGTGATTTGACTAATGGAT -795

AACCCCAGAGCTAGTAAACTGAACACAGAATTAAACCTAGGTCTCCAGAAGCAAATTGTC -735

TCCCACAAGTATATTTACTGCATGAAACTACTTTTTACAAAATATTTCTGGAATGGCTTT -675

TCTCACTATGTGGTCCCAGAAACCCCTATAGCAGCATCTCCTACAGCCTTTATTTTAAAA -615

TGCAGTTTCCTAGGGCCAAGTCTAGATCTGGACAGAGTCCTGGGGGTGCTTTCATATTCC -555

CTCAAACTATAGTTCAGAGTCACAATTTTTTTTTTTTTTTTTTTGAGGAAGATTAGCCCT -495

GAGCTAACATCTGCTGCCAATTCTCCTCTTTTTGCTGGGGAAGACTGGCCCTGAGCTCCC -435

ATCCGTGCCCATCTTCCTCTACTTTATTTGTGGGACGCTTACCACAGCATGGCCTGCTAA -375

GCGGTGCCATGTCTGCACCCGGGATCCCAACCTGTGAACCGTGGGCTGCCAAAGCAGAAC -315

GTGCGAACTTAACCTCTGGGCCACCAGCCGGCCCCCAGAGTCATGATTTTAACCAACATA -255

CTTCGACTGAGTCTTCAGAGATCTCAACCTTAAGTTTCATTTCCTTCCTCTTGATTTTAG -195

ATCAGTTTCACTTTCCAGTTCCCCTTCCCTTTCATAACAGCTCAGAAACAGGGAGAAAGC -135

ATCAGCTGATGCATTCCTATTACCCCCACTTTCCATTTATATAGCTTTTGCAGCACTTAC -75

TTGAGCCAGACTGGAAAGATTTCCGAAGAGCAAATCAGCTTGGATCCCGGCTTTTCAGAA -15

GAGCAGAGAAGCAGATG +3

>IFIT5[Equus_caballus]

TTCTAAACCTCAGCATTATTGGTATTTTAGGCTGGATAATTCTTTGTTGTTGGGGCTGAC -884

CTGTGCATTGTAGGACATTTAGCAGCATCTTGTTTTGACCCAGTTAGATGCTAGTAGCAT -824

TCCTCCAGTGGTGACAGCCACATTGTCTCCAGACATTACCAGATGTCCTCTTGCTGGGCA -764

AAACTACCCTGGTGGAGAACCACTGATCTAGACTGTTCTGCTCTCAGGTCTTTCATGAGT -704

GCCTCCTTTTTATTATTGAGATCTTCCTGCAATTGCCCCTTTCTACAAGGGCTTTTCCTT -644

GTCAAGCATAACTAAAGTACTCTCCCACTCCCACCGTTTAATCCCATTACTCTGGTTCTT -584

TTTTCTTCTTAACACTTATTACTGTCTGGAATTATGTTGTCCATTGATTTGTTTACATGT -524

TTATTTTCTGTCCACTCCGTTTGTTCCCTCTCCCAGAGTGGAATTACCATGAAAAAAGGG -464

CCCCTTTCTATCTTTTTACTATTCTATCCACTACAACTAGAAAATTGCCTGGCAAGTATG -404

AGTGAGGTGATCCATAAAGCTTGATAGAAATATTACTATAGTTCCCATAAACATTTATAC -344

AAATATATATTGTACATACAGGGAGTAGGGTTCTTTGTGGAGCAAAAAGGGAAATGCACA -284

TACACATAGGACACTGTTCATCTCATAATTAGAGCATTTCCCTCTTGGAGAGAAGCCTTA -224

AGTAATTTTTTCTGTACATTTATTGTGACCTTTAATTATGGCCAATAAGACTAAATAGAC -164

TAAAAAGTCTTTCCTGACTGCCTCACCACCACCATCAGCAAAACAAACAAAACTGGGTCT -104

ATTATTAGGACTTTGTCAGTTGCAGACAGTACAAACCCAATTCAAACTGGTTAAGCAAAA -44

AGAGAAAGAGAGATTATTTGCTTAAAAAAATAGAAGTCCCGTGATG +3

>IFIT1[Canis_lupus_familiaris]

TCTCAAGCAGATTCCCTGAGAGCTTGGAGCCTGATATGGGACTCAATCTCACCACACTGA -954

GATCACGACCTTGGCCAAAACCAAGAGTCAGTTGCTTACCCGACTACACCACCAAAGCAC -894

CCCAGCCCTTACTATTCTTTAAGCCGATTAAACAGGGCCACTGGCATCTAGGGAACTGGA -834

CAGTGCTCCTGCACATCTATGAGAGTCATATCTGACAAACAAGAACACTTTAGACCACTA -774

GCATAGAGAGCATTAAAAGCACTGTCATTAAAGCCTATAACGTGTTCTGTTTGAAGTCTC -714

AAGATGCTAGCACAGCCTTTTTCTCTGTGCCAGTGAACAAGGAAGAGGTTATAGGCTCTT -654

ACTGTCATTGGCCATTCTCAGGAAGGGTTTCAGTTAGCAATAATCGTGCCTCAGATTAAC -594

CTCATTGGCTACGATACTGCCACTGCGCAAAGCTCATTCTCAGGAAGTGTATGCAAGACA -534

AAGGCAAAGAAGAATTAGTGAAACACAACTTCTCAGATATTAGCAGCAAATCTGGGTTCA -474

GACTCCAGTTTAGAAATATATGCATCATTTTATTTATGAAAGGAACACCTTCTGTTTCCA -414

GATGAGCAAACAGAAGCACAAATCTGGGGCTTGAGCTTGGCAAGTTCACACAAGCCAGTA -354

TTCACCAGCACTGGCTCCAAGCTCAGTGGACTCTGGACACCTGGCAACACCCCATCACCA -294

GCTCTCCGTGAGGTCATTCAGAGCCTGAGAAAGGAAGTAGCCCATCAGTGAAACTGCTTT -234

CACCAGCCTCTTAACAGTCTCCCAAAGGGAACACAGAAAGCAATCCCTGTTTCATTTTCC -174

TCAAGCTGGAACCTTGCTAACTAGTTTCAGTTTCCACTTTCGTTTTTATCTTGGTTTCCA -114

GTTTAACTTGGTAAAAACACGCCCATAGGTTACATCATTAACTCTTTCTCTTATAAACCC -54

CTTTGTTGGGGCCAAGTCCTTTGCACATCCTCCAGACTGGCTGGATCTCAGAGGAGCCCA +7

TCAGCTCAGCAAAACACTGCAGAACCGCTGTCTAATTCACAGCAGCCATGAG

>IFIT1B[Canis_lupus_familiaris] TCAAATGACTATGTATTATACCTGAAACTAATATAATATTGCATGTTAACCATACCAATA -984

AAAAAATAATGTTTATGCAAAATAATTTTATGCGCAAAAATTCCAAAGCCATACAACTGT -924

CAGTACAAACAGCCTTTCTAACCTTTATGCCAGTACCTGCTTACATGATGGAGCTTTAAC -864

CTCCTGCACTCGGAGCACACAGCCCTAAACTATCTGAGATGGTATGCGTCTGGGAAAGTT -804

CCTACCATCAGTTGATTCTGTCAAATGACAAGTCTCAGATGTGCTTCCTAATAACTCTAA -744

TGGCTTTTTCACTCCACATAAAACAATGTTTGGCTGATAGCCACTCTTCCCTTTAAGTGA -684

ACTTATGCTTCCACAGTGGGCCAGCTTAGCCCTAGTTTCCTTCCTTCTGCTCCCTGGGTA -624

GTTCCACTCTCCTCCATACTCTCATTTTGCTTAGGGAATTTTACTGCCTACTTCTATCAT -564

CAAAGCATACTAAGGAGTGGAATTGGGATTCTCTATATTTGGGCAGTCTTGAGACATGCT -504

GAGTTAGGAAGAACACATGGATCCAAACCATATCCTGTGCTTATAAGATGTTAAGAGGGC -444

CAGTACATCGTTCCGGGAATTAGGAATGGACAAATGTGGTCTACCACAAATTGCCTAAGT -384

CCCTCCTTGGATGAGAGATACAAAGATGACTTTAACTCATCCAGAGAGGGATCTTGTATG -324

ATGTTCTTCTTAAGCACACACTTTTCCTTAAAAAAAAATTTATATTTGAATGCATAAAAT -264

AGATACATTTCATGTTTCATATTTCTTTTTTTGGATAATCCTTCCTGTTATATAACCTTC -204

AACTTTAGAAATGTCAGGACACTTCTCTAGGTGCTGAAGCAAAATGTAGAAGTTCTCTGA -144

AGAGAAAGAAGAGCCCCAGACATCTGGGAGCCAGCATGACACTAACAGCTAGGCTCTGGT -84

GTTGCTGGACAGTGACCCACAGCAAGAACCTGTGTTTTTCTGTAGCACATTACATTAACA -24

CCAGATGAGGCCACCCTGACACCATG +3

>IFIT2[Canis_lupus_familiaris]

GAGGGATGGATGGTGGACCCTGAGAGGAGCTAACATTCCAGAAAAGGCAACATCAAAAGT -991

GTCTCCTCTGAGACACCTGGGAGGCTCAGTTGGTGAACCGTCTGCCTCGGCATGATCCTG -931

GGGTCCTGGGATCCAGCCCCCCACAGGGCTCCCTGCTCAGGGGGGAGCTTGCTTCTCCTT -871

CTGCCTGCTGCTCACCCTGCTTGTGCTCCCACTCTCTCTCTCTCTCTGTTAAATAAATAA -811

ATAAATAAATAAATAAATAAATAAATAAATATATATAAATAAAATCTTTTTAAAAATTTC -751

CCTTCTGGCCTCTTTGGTTAATGTTGGTTAATGACTTTAGCTATTAATTATAATAAAAGG -691

TAGCATGCACTTCTTATGTGCCAGGCACTATATTAAGGACATTTCATGCTTTAACCGATT -631

TGATCCTCACACAGCACTATTATGAGTTGGGTAGTAGTAGTGGGATCAGCATTTCACAGG -571

AGGACAGACGAAAGCACGAGAAACATTCAGTAATTTCCAGTGTCACAGCACATACACAGA -511

GCCTGGGTTCAAATCCAGACAGGGCATCAGAGCCCATGGTTCTGATCATGTTTTCATTCA -451

TTCAGCCTCTACCTTCTGAATGCCTGTCATGTGCCCCATACTAATGCAGGGACTAGACAC -391

TCAGTCCTTAATGAAACAGCCCTCTTCCTTTCAGGGGCCCACAGACCACAGGGTAATAAG -331

GGTGAGCACAGGGAGGATGGGTAAACATTGTGGTAATGAAGCAAGCTGCTTGGTGTTTGG -271

TACCCGCATCCTTCTCCAAGCTGGGCTCCCTTGATGTCAGCTGATCTAGGACATCATTAC -211

AGCCTGAAGGAAGAAGTTCTGGCTCACTACAGGGTGGGGGACACAGGGTGCTGGAGGCGG -151

GGCTCTTACTCAAGATTCATTTCCTAGTAGAGCCAGACCAGTTTTACTGTCCAGTTTCAG -91

TTTCTGTTTCCTAAAGTGAACTGCAGGAAAACAAGGAAGAAGCGCCAGGAAGTGCTTGCA -31

TATACAGGTTTCCTTAGTGTCTCTCTCGGAGGCAGA +6

>IFIT3[Canis_lupus_familiaris]

TCCCGCGTGCGCGCTGCAGCCCTTAGGGAGCTCGGCGCACTCTCCCGGGGCGCAGTTGCT -998

GTTACTGTCCCCGGAGCCCGAGGGCATCCCCGCCCTCCTGGGTCCTGCTCCACCTCCCCG -938

CGAGCCCCTTTCCCCCGGGGAGGTTGGTGCAGCTCCTGCTCCTCCGGGACGGGGCTCTCC -878

TGTCCTGGGGACACTCGCCCCGGCCTCAGCCCGGCTCCTCGCGGGGCCCCTCCCCCTTGG -818

AGGCCTTTGTTTCTTAATTTCTTTTTCCCCGTCTTCCTACCTTGATAGAAGCGCGAACTC -758

TTCTCACTGTAGCGTTCCAGGTGGTCTCTCTTTAAATCTCAGGCCAAATTCGTAGATTTT -698

CAGGATGATTTGAAGGTTATCTAGGTAATTTGGTGGGAACAGGGTGACTTGGGGACCCTA -638

CTCTTCCGCCACCTTGCCCCTCCTCCTCCATTAAGGCCTTTTCTTCAGATTCTACCTGAA -578

GGTTAAGACAGGATGGACTCTGGGATGGAGAACTCTTGGCTGTCCTGTCATCAGTTCTGC -518

CTTGCACTAGAGCCATCAACTTTGCTCAGATCAAAAGTAGGCAGGTAAACATAGTGGCCT -458

TACAATGTTCTACCCCAACTGCTTTTTCTGTGACTTTGTATATTTGTCAATAGCAAAACC -398

GAGCTGGATCTTTAGCTTTATTCCCATTTCTTTATACAAGAGTCTTTATATAATTGGACA -338

TATTTTTTGGTTTTCCTCACAGAGAATTTTAACCCTCTTTTTATTCTTTCTACAACAATT -278

TCTACATATACTTAATTTTGCCTACTTTTTTCTCTACTTTCCAGTAAAATAAAGGTTAAA -218

GATTCTCCCTACTGCTGGTTTAAAATGTGTGTGTGTGTGTGTGTGTGTGTGTGTGTACAC -158

ACAAAAAAATGAAAAAGAGAGATTTGTTATCAGGAGTTGACTCACATGATTATGGAGGCT -98

GAGGATCATAACCATGGTCCACTGTCCACAAGCTTTAGACCCAGGAGAGCCCATAATGTA -38

ATTCTAGTCAGACTCCAAAGGTAGAACTAGAGGTATAATGCC +5

>IFIT5[Canis_lupus_familiaris]

CTTCTTCCTCTGCCTGTGTCTCTGCCTTTCTCTGTGTGTCTCTCATGAATAAAATAAAAT -1131

CTTTTTAAGAAAGTGTATTAAACAAGGCACAGTTGGCAAGGGCACAGAAGGCGAGCAGTG -1071

TTATACAGCTCTAGATAGTTGTATAAATCTTTCAGCAAAGCCTTTCAGCACACAGCCATT -1011

AAATTTTATTTTATTTATTTATTTATTTATTTATTTATTTATTTATTTATTTATTTATTT -951

ATTTATTTATTTATTTATGATAGGCACACAGTGAGAGAGAGAGAGAGGCAGAGACACAGG -891

CAGAGGGAGAAGCAGGCTCCATGCACCGGGAGCCCGACGTGGGATTCGATCCCGAGTCTC -831

CAGGATCGCGCCCTGGGCCAAAGGCAGGCGCTAAACCGCTGCGCCACCCAGGGATCCCCA -771

GCCATTAAATTTTAAAATGTATATTCATTTTGACTCCAAACTTTCTCTAACTTTACCCAA -711

CGGGAATAATGACATGGGTGGGATGTATAGTAGCGCACCTGTGTTGTTTGTTGAATCAAG -651

ATGGGAAGCAGGGACTTCTTTCTGCTTAGGATGCAGAGAGATATAAAGAGCATCGCTGCA -591

GCCCTTACAACAAAAATAACGCAGAACAATCTATTGTCCTTCTTTTTAAAAACAATTTTT -531

TTTTTTATTTATTCATGAGGGACACAGAGAGAAGCAGAGACAGAAAGGAGAAGCATGCTC -471

CTCACAGGGAGCCTGATGCAGACTCAATCCTCAACCCCTGAGCCACCCAGGCGTCCCTCC -411

TTTTTTTTTTTTTTAAGATTCTATTTATTGGGGAGAGAGAGGGAGGGTGGGCGCACGAGC -351

AGGGGAAGGGGCAGAAGGAGAGGATGAAGCAGAGTCCCCACTGAGCAGGGAGCCCCAGGC -291

TCAATCCCAGGACCCCAAGAGCATGGCCTGAACGGAAGGCAGAGGCTAAATGACTGAGCC -231

ACCCAGGCGCACCTCTCCTGTCATTTTATTATTTAAAAAAAAAAAAAAAAAAAGGGTCGA -171

TTGAGTGGCACGCATAGTCGGCGAGTTAGGTGAATGCCCAGACCCGCGTCCAGAGGCCCC -111

GCTGCAAATCCCACAGCGAACCACCCTCGGTTCCGCAGACTCTTCTCCACGCTCTTACAG -51

AAGTTTCTGTTTCCTGGAGGGGACGCGCCTAGTCCGGACGCAGAGTCACTATG +3

>IFIT5L[Canis_lupus_familiaris]

ATCCAGTGAGAAAAATAGTTAAAACAAGTTCGCAGTCGATTAAAGGTCAGCTTTCTCCAT -953

GAAGAAGTTTGGAACTTAATTTGCCAAAGTGTTCCAAGCAGTTCTCTTGTTTTATGCCAT -893

TTGGTTTGAAGCACTATTAATTCAGTCATTTGCTGGTATTGATCAAGAAACATGAGAAGG -833

TTGCTATATGATTTGAGAGACCATCAATAACAAACACCTTTGATTATGACATTTTATTAA -773

AAGAAGTTGCATGCCATATAATATTTTTATTTTGAAAAATATTTTATTTACTTATTTTTG -713

AGAGAGAGAGAGAGAGAGTGAGCCTGAGCATGGGGATGAGCACGGGGACAGGCAAGAGAA -653

GCAGACTCCACGCTGAGCAGGAAGCCCAATCTGGGGCTTGATCCCAGGACTATGGGATCA -593

TGACCTGAGCTGAAGGTAGATACTTAATCAACTGAACCACCCAGGTGCCCTCACCAAGTA -533

CTATTTTTAAAAGGAAAAAATTAAGGAAACAATGGTACTGTTTTTTCATTTTTGTATTTG -473

TTTTGGGGGAAAATGTATTAATTTCCTATTGTGGCTTAATTAATTACCACAAACTTGTTA -413

GTCTAAAACCATACAAATTTATCATCTTACAGGTCTGGAGGTCAGAATCGGAAATGAGTT -353

TCACTGGACTGAAATCAAGGTGTTGGCAGAGCCAATTGCCTCCAGAAGCTCTAGAGGACA -293

ATCCTTTTTGTACTTTTTCCAGCTTCTAGAAGCTTCCTACATTCCCTGGTAGAGACCCAT -233

AATCCACAAGATTATAAATCCCCCAGGCATTTATAGAACTATCTGGTAGCGTTTGGATTT -173

CTACTGAACTCTAAATTGCAGGGAAGAAATTGCAGGGAAGCAAACTTTGTATCATTAAGG -113

GCAGACATCAGAGTCAGGTAGAAGCCATCTTCCCAACTTGTTTATCACTAAAACAAGAGA -53

CTTATTAGATTTAACAAGAATGATATTCTAAAGTGCTTGGTTTGGCGCTAAAATGAGTTT +8

AACC

>IFIT5[Gallus_gallus]

GCCCAGCTGTCTTGTCCCACTCAGTGGGTTTCGAGACAGGTGAATCTTTTTCTATCTCCT -997

GAGTCACCTCCTCTAATCAGCAGGGCTCTCTCCTGCGATGGAGTCATCCAAGACTCAGAG -937

GAAAAATACCAATCAATTTATAGAGATCTTTCTAGTAGTAACTAATACTCTACTCGAATG -877

TAAAATTGGTTAAATTCCTAATAATGATTAATAAGCGCTACAGGTAAGGCAATATTAGGC -817

AACGGTTACTACAAAGAGTAAAAGATAAATGATAGATAACAGGGAAAGAGAGAGACAAGC -757

AGACATACAGATATAGAAATACAACAAAGCGAAGCAGTTCACCACTCCTCAGTCCAGCCA -697

TGTCTTGATGGTGCTGATCTTTGGTAGTGGTGGGCTATCCATTTGTTGTGGTAAGCTGTG -637

GACAAATGGCAAAAGTACGTACCCTTGACTTATAGCCTTGAGTGGTCCAGCCTGCTCTTT -577

TGGAATGACAGTTTCTGGCTTTGGGGACCTTCATTGGATACAATGGTATCATCTCCCAGT -517

CTCTGGTATCAAGCTGGAAACATTTGTTTCCAGGCCAAGTCCCCAGACAGAAAACACTCC -457

TGGACCCTCCCATTCAAAGGTAAACAGCTCCCAAGAAACACTTTTGAACGTAAAACCATC -397

CACAAAGTGATTCAATGCTGTCACGTTGATTTGAGACAATCCATACCTTACCAGTCTCAG -337

GCACCAGCAAACACCCAGCTGCCACCCAATACAGAACAGCCACTCAGCTATGGGCTGCAC -277

TAGCTAGGTAGTTCTCAGTGCTAGCAATTTGAGGTTAAATTCAGATATGAAGCTGCTAAT -217

AGCTGTTACCATGGGCTCCACAGCACAAGTCAATGTGTGGCATAAAGCCAAAATGAAGGA -157

AGCAGAATGACTCATAAGTTTCTGTTTCCCAGACGGTTGCCTTGTAGCTCCTCCCAGACG -97

CAGGAAGCTTGCTTTCACTTTCTGATTCCCAGTTTCGTACACACCAATCACGTGAACGTT -37

AGAGCACATAAAAGGCAACCCACAACTGCAGGGAGCAGCTCTTGAGCTTCAA +16

>IFITB[Taeniopygia_guttata]

GGGCGCCCGGCCCGGCGTGATTCAGCGTTTCCGCAGCAATGCTGTGGGGATCCTCCCGCC -1102

CACCGGGCTGCATTTCCAGGAGCCTTGCAGCTCCGGAGCCCGCGCAGCGCCGCCCTGCCG -1042

CGGTTCGCGTTGTGCTCCGCAGCGGGCTCCGAGCAGAAAGCGGCGGCAGCAGCCGCGGGA -982

GGGCGGCGGTGCCGCTCCCCGAGGCCGCAGCCAGCATCCCCGGCGTGCCTGCGGTGCCGC -922

TCCCCGAGGCCGCAGCCAGCATCCCCGGCGTGCCTGCGGTGCCGCTCCCCGAGGCCGCAG -862

CCAGCATCCCCGGCGTGCCTGCGGTGCCGCTCCCCGAGGCCGCAGCCAGCATCCCCGGCG -802

TGCCTGCGGTGCCGCTCCCCGAGGCCGCAGCCAGCATCCCCGGCGTGCCTGCGGTGCCCG -742

GCACCTGCGCCCCGGCACCGCCCGTCCCTCCTGAGCGCCCTGCAGCGCCGACCGCAGCCG -682

CTGCTGCTCTCACACACATCATCCATCCGTCATCTGCCAGCACTGTCTGAACGTAGCCTA -622

CCTTATACAAACAGGCTTCAGTTGCATGCACCGTTACCGTCACAGCTTCCTGCAAACCTG -562

TGGCATTTTCATGCTCTGCTGTAACTGATTTGCAGGCACTGCATTGCACAGCTGTAATCT -502

CCCAGAGATGCTTGTCTGTAGTCAGGGGATGTGTGACCGGGCTTGCAAAAACTTGGACCA -442

AATTAGATCCTTTAAAATGAATATTCTGTTACTCTGCCCATTACTGTGGAATAATATCCA -382

TAGAATAATACAGAAAATTGATACTAAATATGCAGTGCTAGAGCAAGAGGGTGTCAAGAA -322

CAGAAATGTTTCTGTTTATTTAGAAAGCATTAAGATTTTAATTGAAAAATCATAGAAGTT -262

TTCATAGCATACCTGTGTTTTGATATGTGGATATGTCACTAGATACAGAGATACTATATG -202

ATACTTTGCATTCCTGGATGCTATTTCTTAAAACTGATTAATTTTAAATAACTTGGGTGG -142

AAGAATTATTGCTCAGTGATGCACTTGAGATTTTTTTGTCAAATTAATTACAGGTTACTC -82

TTCCCTGCCAAGGGAAGACTCAAATTATAAAAGCCACAACTGCTGCTGTCAAACATAGTA -22

ATTATATGCTAACCTAGTGTCATG +3

>IFITA[Ornithorhynchus_anatinus ]

GGTTACTTGGGTTCCTCAGGACCTAAAATGGAAGCAGAAGACAGGAATGCCAAGGTTCCG -929

GGGTTCAGGAAGATCCAGGCCCAAGGTTTTTCTCTACTGGTATGGGAACACCTATTCCCA -869

GTGGCAGAGAGCTCCCATTACATGCACCTCCCTAGCAAAGAATCTGAGCCTTGTGATTAG -709

CACAAGAATAACTGTGGTTGCTAAGTTCTCCCCCTTCTTTTGGGTGCACCTACCAGTGAA -649

CAGACTTGATGCAGATTAAATGATTTCATTATTTCTACATTCATATCCCTTGGTCCCATC -589

CCTGCCATGACATGGAGTGCCATGGACCTTTCTGAGATGTTCCTTTTCTTCCCAAAGAAA -529

TAGGGCATGGAGAGTCATGTAGCTTTAAGGGTGGTTGGCTAAAGCCCCGCACTAATCCTT -469

GGGACCCCCATCACCTAGCTTCAATCATTTTCAACTAGAGGCTTTCAGTACCCCCTTACT -409

TCTGTGTCTCCCATCTTAATGACTCCATTTCTTCTCATTAGTAACAGTAATAATGATGAT -349

GATGGGAATGTGTCTACCAATTCTGTTATATTGAACATTCTCACATATTTAGTATAATGC -289

TCTGCACACAGTAAGCACTCAATAAATGCTATTGCTTAATCGAGTGATTGGTCTGTATCT -229

TTTCTCCAGCAGCTGATCTTTCGCCTACTTCACAAGCCTGGATTTCCATTTTACACCTGG -169

CCTCCCACCTGACATCCTATTTGCAATCCTGGCTTTTTTGCCTCACATTTTTCTTTGCCT -109

TTCTGGCCTTCTGTCTTACATTCTTCAGCCGGACTGGGTTAACCTAGAACCTGCACCCAG -49

AGTCTCCTGGTGTCCTTCATTCCCCCAAGGCCTTTACCCACGGTAACCATGTGTAGCACT +12

>IFITB[Ornithorhynchus_anatinus ]

GGCTCCTGGTAGGTGTCTAATAAATACCCTCATGATTATGTGTGCTCTCAAGTACAGTGC -808

TCTGCACACGGAAAGCACTTGATGAATAATTGGAGAAGGGTAGCAGAGCAGTCTTGGTAG -748

GATATGGGCCAATCTTTCCAAGACTGGTCTTAGACATGGTCCCTTATCCCCCAAGGGGCT -688

GCTATCGGGGCAAATATGGTCCTTTAGATTATAAACTCTTTGAGGGCAGAGACCACCTAG -628

TAATAATAATAATAATAATAATGGTATTTGTTAACCACTTAGAAGCAGCGTGGCTGAGTG -568

GAAAGAGCCTGGGCTTGGGAGTCAGAGGTCATGGGTTTGAATTCCGACTCTGCCACTTGT -508

CACCTGTGTGACTGTGGGCAAGTCACTTAACTTCTCTGTGCCTCAGTTACTTTATCTGTA -448

AAATGGGGATTAAGATTGTGAGCCTCACGTGGGACAACCTGATTAACCTGGATCTACCCC -388

AGCGCTTAGAACAGTGCTCTGCACATAGTAAGCACTTAACAAATACCAACATTAATTAAT -328

TAATTATGTGCCAGGAACTGTACTGAGTGCTGGGGCTTATTTTGACAAGACGGTTACAGT -268

ACGGGCCTGGGCAAACTACCCGATTTGGACGGGGAAGCAAGTTTGACTTACCAAGGGATG -208

GATGTTCGGGGCCCAAGATGGCCATGTTAAGCGCTACTTGTGGGCACTGAATGTGTCGAT -148

TCTCTCCTTGGTCTGCTACTCGCCCAAACTCTTACTGAATCGCTCTGAACTCGTTACCTG -88

CCCCAGAAATATTACTTACTATGGTGCACGGCACTGTCTTTAATCAGTTAGGACACTTCA -28

ACCAAAGAAGATAACAGAAGCATCCTCATGCCTCACAGTCCAGCGTATGGCCTGCAGGGA +3

CTCCAGCCCAAAGAGGCAGAAGTCTTACAACCGCATAGGTACTTCGAAAACGTCAAGGAT +63

>IFITC[Ornithorhynchus_anatinus ]

TCATATTCTAAGAATGAGAAAGAAGAAAGGGGTGGATTGGGGGTGGGGGAGAGGGTACGG -751

ATGGTGCCAGATGCATAAGGAAGTCAAACAAAAATACAAAAGAGAAAATTGTGGTATTTG -691

TTAATGGCTTATTATGTACCAAATACTAATCACTGGGGTAGATACAAGACTATCAGTTCA -631

GATGCAGACCCTGTCCCTCATCGGTTTCATAGTCTAAGGATGAGGATTTCTAAGCTTCAA -571

AAGTCACAGCTGCAACTAATCAAGTCCTTCCCCAAGACGACTGGCAGGATTGACCAGAGC -511

CCCAGCATGATGGCTTAGGGGCAGAGAGAAAGAGAGATAGAGAGACAGAGAGACTGAGAG -451

AAAACGAACCTGGGTAAACCCTTCTGAAGTGACTGAAGAAAGGCTATTTCTACAGGTGAT -391

CAATAGAGACTATTACCAAGAGATCCTGTAATCACCAATCAAGTGTTCAACCTCCTGGGA -331

TTCACAGTAAAGAAAGAAAAATCTGCATCCTTCCCCAAAATTATTCCTTGGTCAAATTTC -271

CAGAAAATTAAACCTCCTTATTCTCAAACTATGAGGTCATCTGCCTTAGAAAAATGTAGT -211

TCGGCCCTCCGCTGAACAAAATAATAAAGTTTATTAATCTTATTCCCAGATCTTTGAGAT -151

CTTCCACTTCCATACATAACTGTTCTTCTAGAATCGCCTAGAAAGTTTCAAGTTTCAAGT -91

TGCAAGTTGCAAGTTTCTATTTCCTAGAATGCGATGCAGAGTTATGAAATGATTTCACTT -31

TCCATTTCTCTCATTGGTACCAGAAGTACGAAATACCAAGAAGTATTGGTACCAGCTGAT +30

GCTTTTGTCGTATTTAAACCCCAGTTACTCTGACTAGTCAACCCACTTTCAGAGTCACCT +90
