# Supplementary material for: Lineage-Specific Expansion of IFIT Gene Family: An Insight into Coevolution with IFN Gene Family
Source: PLoS One. 2013 Jun 20;8(6):e66859. doi: 10.1371/journal.pone.0066859 (PMC3688568; doi:10.1371/journal.pone.0066859)
Supplement: Table S3 — Primers for expression analysis and luciferase assays of zebrafish IFIT genes. (DOC) [file pone.0066859.s006.doc]

STable 5

Primers for expression analysis and Luciferase assay of zebrafish IFIT genes

| Primer name | Sequences (5’-3’) | Usage |
| --- | --- | --- |
| DrCh1732RT-F | ACGCCCAGGAAATCCAAGC | RT-PCR |
| DrCh1732RT-R | TTGGATGAAGCCCAGCAGG | RT-PCR |
| DrCh1722RT-F | TGAACTGTCACAAACGTGGCTG | RT-PCR |
| DrCh1722RT-R | GACAAATTGTTTACACTTAGGAGGC | RT-PCR |
| DrCh1712RT-F | AGACCACTGCTGTTCACCGC | RT-PCR |
| DrCh1712RT-R | CCAAATAGAAGTAGACCCAAGCC | RT-PCR |
| DrIFI56mRNA-SF: | TGATCTTCAAGCATGGCACAGG | RT-PCR |
| DrIFI56mRNA-SR: | GTCACCTCTGCTAACACTGTTAGAC | RT-PCR |
| DrCh1255RT-F | CATTTGTCTTATGCTCAGTTCCAG | RT-PCR |
| DrCh1255RT-R | GTCTGGAGTGTTCCCATACCTTTC | RT-PCR |
| DrCh1245RT-F | CCTGAACCCAGATGATGATGCTC | RT-PCR |
| DrCh1245RT-R | GCTTTGTCAAACTGTTCCTCTGCC | RT-PCR |
| 3ch1235RT-F: | TGGGTTGATGCTAATGGCC | RT-PCR |
| 3ch1235RT-R: | TTCCTGCCGAGTCTGTTCC | RT-PCR |
| 2Ch1225RT-F | TTTACAAGAGGTACGGGGAGGC | RT-PCR |
| 2Ch1225RT-R | GAGGTTGTCAGGCTGGTGGC | RT-PCR |
| DrCh121RT-F | CCCTACACAGCTTGGACGG | RT-PCR |
| DrCh121RT-R | GCCTTTTGAAGATAGTCCAGTGC | RT-PCR |
| DrCh55RT-F | CTTTCATCCAGTATCTCAACGGG | RT-PCR |
| DrCh55RT-R | GCCCTTTTCTCTGAGCACTTCC | RT-PCR |
| Ch5SIFIT5Prom2-eF | TAGGGTACCTGAACACCATGCGGG | Luciferase assay |
| Ch5SIFIT5Prom2-eR | AACAACTCGAGCAACCAACCCACC | Luciferase assay |
| Ch121SVHSVProm-eF: | TTTTGGTACCTATGAATCTGCCCC | Luciferase assay |
| Ch121SVHSVProm-eR | TAAACCTCGAGTTCAAACAACAGG | Luciferase assay |
| DrPromoterS-F | GATTCGGTACCATGTAGCCTTCAG | Luciferase assay |
| DrPromoterS-R | ATAAGCCTCGAGTTCTTCAGCGTG | Luciferase assay |
| Ch171SIFIT2Prom-eF | TGGGTACCATTGGTGGTTATTAGC | Luciferase assay |
| Ch171SIFIT2Prom-eR | TTCTCGAGACTTACTCCATTGCGG | Luciferase assay |
| Ch172SIFIT2Prom2-eF | TGTTTGGTACCTGCTTCTGATGGC | Luciferase assay |
| Ch172SIFIT2Prom2-eR | AGAGACTCGAGAATTTAGGCAGGC | Luciferase assay |
| Ch173SIFIT2Prom2-eF | TACGGTACCGAATACGTCAGACCG | Luciferase assay |
| Ch173SIFIT2Prom2-eR | TTCATCTCGAGTCCAGACAGGCAC | Luciferase assay |
